# Hedgehog signaling in tissue homeostasis, cancers and targeted therapies

**DOI:** 10.1038/s41392-023-01559-5

**Published:** 2023-08-18

**Authors:** Junjun Jing, Zhuoxuan Wu, Jiahe Wang, Guowen Luo, Hengyi Lin, Yi Fan, Chenchen Zhou

**Affiliations:** 1 State Key Laboratory of Oral Diseases and National Clinical Research Center for Oral Diseases, West China Hospital of Stomatology, Sichuan University, Chengdu, 610041 China; 2 Department of Pediatric Dentistry, West China Hospital of Stomatology, Sichuan University, Chengdu, 610041 China; 3 Department of Cariology and Endodontics, West China Hospital of Stomatology, Sichuan University, Chengdu, 610041 China

**Keywords:** Cell biology, Cancer

## Abstract

The past decade has seen significant advances in our understanding of Hedgehog (HH) signaling pathway in various biological events. HH signaling pathway exerts its biological effects through a complex signaling cascade involved with primary cilium. HH signaling pathway has important functions in embryonic development and tissue homeostasis. It plays a central role in the regulation of the proliferation and differentiation of adult stem cells. Importantly, it has become increasingly clear that HH signaling pathway is associated with increased cancer prevalence, malignant progression, poor prognosis and even increased mortality. Understanding the integrative nature of HH signaling pathway has opened up the potential for new therapeutic targets for cancer. A variety of drugs have been developed, including small molecule inhibitors, natural compounds, and long non-coding RNA (LncRNA), some of which are approved for clinical use. This review outlines recent discoveries of HH signaling in tissue homeostasis and cancer and discusses how these advances are paving the way for the development of new biologically based therapies for cancer. Furthermore, we address status quo and limitations of targeted therapies of HH signaling pathway. Insights from this review will help readers understand the function of HH signaling in homeostasis and cancer, as well as opportunities and challenges of therapeutic targets for cancer.

## Introduction

HH signaling pathway was originally identified for its role in patterning the Drosophila embryo development.^1^ When mutated, the larvae of Drosophila were covered with short barbed spines like a hedgehog.^2^ As an evolutionarily conserved and highly organized signaling, HH signaling regulates numerous processes of vertebrates and invertebrates, such as embryogenesis, cellular development, epithelial-mesenchymal transition (EMT), and varieties of pathological variations.^3,4^ HH signaling pathway plays a vital role in the regulation of homeostasis in various tissues, such as stem cells in musculoskeletal tissue and digestive system.^5^ Moreover, Evidence-Based Medicine has shown that abnormal HH signaling is a considerable driver of tumorigenesis and malignancy.^6^ Understanding the function of HH signaling offers potential therapeutic interventions. Some targeted tumor therapies have entered clinical trials as the mechanism of HH signaling pathway is further explored.^7^ The current review aims to provide a comprehensive background on HH signaling pathway and highlight the recent advances of its role in homeostasis and tumorigenesis. It will also shed light on the available means and potential challenges of targeted therapies for HH signaling pathway-dependent cancer.

## Hedgehog signaling

### Introduction of Hedgehog signaling pathway

The initiation of Hedgehog signaling is based on the processing of Hedgehog ligands in secretory cells, where a dual lipid-modified amino-terminal (N-terminal) polypeptide of the autocatalytically cleaved precursor protein is formed.^8^ Signal reception is achieved by conserved receptors on the cell membrane, including the 12-pass transmembrane protein Patched (Ptch) and the 7-pass transmembrane protein Smoothened (Smo). In the absence of hedgehog ligand, Ptch inhibits the activity of Smo causing pathway inhibition. When the hedgehog ligand binds to Ptch, the inhibition of Smo is lifted, thus continuing to activate downstream signaling.^9^ In addition to this, the activation of hedgehog signaling requires co-receptors, which can be involved in the inactivation of Ptch.

In mammals, hedgehog signaling activation elicits a transcriptional response in the nucleus of the recipient cell that is mediated by glioma-associated oncogene (Gli) transcription factors.^10^ Gli proteins include an amino-terminal transcriptional repression domain, a zinc finger DNA binding domain and a carboxy-terminal transcriptional activation domain. When transcription is not activated, Gli is processed by protein hydrolysis into the form of a repressor with a truncated carboxy-terminal activation domain, which inhibits the transcription of the target genes.^11^ In addition, Suppressor of fused (Sufu), a negative regulator of Gli, is essential for pathway activity in mammals.^12^

A particular aspect of mammalian hedgehog signaling is the transport of signaling molecules at all levels in the primary cilium.^13^ The primary cilium consists of ciliary membrane, axoneme and basal body. The presence of intraflagellar transport (Ift) proteins on the axoneme mediates the transport of signaling proteins, and this transport mode can regulate the concentration of the corresponding proteins within the cilium.^14^ In addition, primary cilia form compartments that are separated from the rest of the cytoplasm of the cell and thus provide a unique intracellular environment for pathway components.^15^

### Components of Hedgehog signaling pathway

In this part, we will briefly introduce the signaling process to understand the mechanism of HH signaling pathway in tissue homeostasis and tumorigenesis. A large body of researches have reported the core components of HH signaling pathway. This cascade includes extracellular Hedgehog ligands; cell surface receptors and co-receptors; Gli family transcription factors (Gli1-Gli3) containing zinc-lipid structures and numerous intracellular and extracellular co-regulators.^16^

### Extracellular Hedgehog ligands

Hedgehog ligands are paracrine signaling factors that can mediate communication between cells up to hundreds of micrometers away.^17^ Three soluble extracellular Hedgehog ligands have been identified in vertebrates, including Sonic Hedgehog (Shh), Desert Hedgehog (Dhh), and Indian Hedgehog (Ihh), which have distinct distributions in different developmental stages and tissues.^18,19^

### Shh

Shh-triggered signaling is well documented for its critical role during embryonic and postnatal developmental processes. For example, Shh forms a ventral and dorsal concentration gradient in the neural tube, thereby directing neural progenitor cell fate in time and space as well as acting physiologically as a morphogenetic agent.^20,21^ Moreover, Shh directs the growth of post commissural axons along the longitudinal axis of the spinal cord, acting as a major mediator during central nervous system development.^22,23^ Collectively, Shh plays a vital role in the developmental regulation of various tissues such as skull, adrenal cortex, hair follicles and sweat glands.^24–27^

**Fig. 1:**
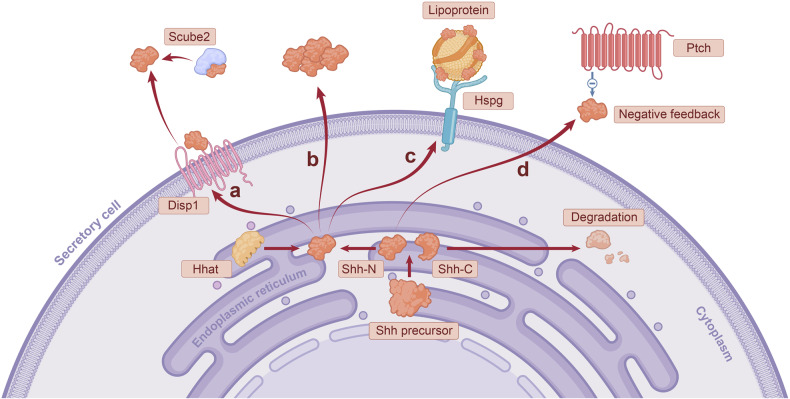
Activation and spread of hedgehog ligands in secretory cells. The three vertebrate hedgehog ligands Shh, Dhh and Ihh have similar secretion processes, differing slightly in the tissue distribution of the three. The figure shows the secretion of Shh as an example of the secretion process of hedgehog ligands. Activation of Shh occurs in the endoplasmic reticulum (ER). The Shh precursor is first processed into two parts, Shh-N and Shh-C. The carboxyl terminus of Shh-N is bound to cholesterol by a protein hydrolytic cleavage process, while the amino terminus of Shh-N is bound to palmitic acid mediated by Hhat. In contrast, Shh-C is degraded after being transported out of the ER. There are several mechanisms for the diffusion of Shh-N out of secretory cells after activation: **a** Shh-N is mainly transported out of secretory cells by the synergistic action of Disp1 and Scube2. **b** The polymerization of monomer-activated Shh-N into multimolecules facilitates the diffusion of ligands. **c** Hspg is localized to the secretory cell membrane, which recruits lipoprotein. Shh-N is loaded on the lipoprotein as a "passenger" for long-distance transportation. **d** Ptch1 on the surface of the receiving cells has a negative feedback regulation on the release of Shh-N

**Fig. 2:**
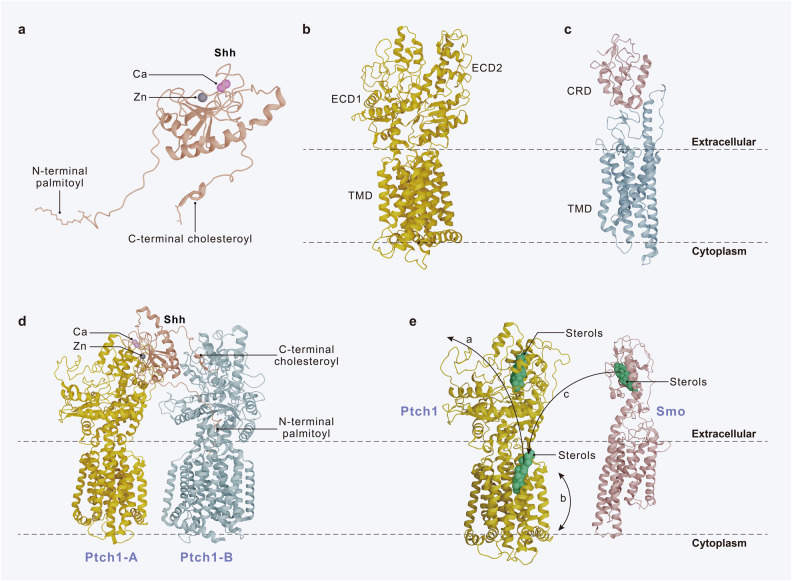
Three-dimensional structure of Shh, Ptch1 and Smo. **a** The structure of the activated Shh. **b** The structure of Ptch1. **c** The structure of Smo. **d** The structure of the 1Shh:2 Ptch1 complex. Ptch1-A binds to Shh at the interface of its calcium and zinc binding sites, which drives Ptch1 degradation. Ptch1-B binds to the N-terminal palmitoyl and C-terminal cholesterol modifications of Shh and anchors to the core of Ptch1 protein, which decreases the protein activity of Ptch1. **e** Mechanism of Smo inhibition by Ptch1. Ptch1 regulates the binding of sterols to Smo, and hence inhibits the activity of Smo. Three possible mechanisms are shown by the black arrows. **a** Sterols move from the outer leaflet of the membrane to ECD1 via Ptch1, which depletes the sterols inside the membrane. **b** Ptchl inhibits Smo activity by decreasing its accessibility to sterols from the inner leaflet of the membrane. **c** Ptch1 accepts sterols from CRD and transports them to the membrane, thereby inhibiting Smo activity

Activation of Shh following synthesis on the endoplasmic reticulum of secretory cells involves lipidation by cholesterol molecules and palmitic acid moieties (Fig. 1).^8^ The secretion of activated Shh serves a crucial role in regard to its biological function. Shh was first cleaved into a 19 kDa Shh amino-terminal polypeptide (Shh-N) and Shh carboxy-terminal polypeptide (Shh-C). Then, Shh binds cholesterol to the carboxyl terminus of Shh-N through a protein hydrolytic cleavage process, whereas, Shh-C exits the endoplasmic reticulum and enters the proteasome to be degraded, ending its mission.^28,29^ In general, palmitoylation occurs after cholesterol modification, further increasing Shh activity.^30^ Palmitoylation of Shh is mediated by HH acyltransferase (Hhat), which transfers palmitic acid to the amino-terminal cysteine residue of Shh-N.^31,32^ The activated form of Shh is a dual lipidation of cholesterol and palmitic acid by Shh-N (Fig. 2a).^20^

The mechanism by which the diffusion of activated Shh from secretory cells occurs is more complex (Fig. 1). Dispatched-1 (Disp1), a 12-transmembrane protein, is required for the secretion of activated Shh. Recent studies suggest that Disp1 binds cholesterol molecules of Shh-N and, together with Scube2, a secreted glycoprotein, transports Shh out of the cell.^33,34^ If the cholesterol and palmitic acid of Shh are broken down, Shh is also degraded.^35^ In addition, monomer-activated Shh-N can polymerize into multimolecules, a form that naturally facilitates the long-distance transport of ligands.^36^ Heparan sulfate proteoglycan (Hspg) is localized on the surface of secretory cell lipid membranes, where it interacts with Shh-N and recruits the lipoprotein lipophorin, resulting in the loading of Shh-N on lipoprotein for transport.^37–39^ In addition to the ability of secretory cells to positively regulate Shh secretion, Ptch1, which receives Shh receptors on the cell surface, is able to participate in the negative feedback regulation of ligands, limiting the range of Shh signal waves.^40^ In conclusion, Shh is able to assemble into suitable carriers for transport, and other possible mechanisms that help Shh diffusion deserve further investigation.

### Dhh and Ihh

Dhh and Ihh act more specifically than Shh and show pronounced tissue specificity. Dhh is a key regulator of gonadal tissue development, especially in ovarian granulosa cells and spermatogenesis.^41,42^ Expressed by Sertoli cells, Dhh directly mediates the proliferation and differentiation of spermatogonia through the regulation of downstream signaling pathways. Dhh-deficient animals exhibit abnormal testicular mesenchymal cell differentiation and defective peritubular cell morphology, resulting in sterility in male mice.^43^ The function of Ihh is mostly related to chondrocyte differentiation in the skeleton.^44^ It regulates the differentiation of growth plate chondrocytes through a negative feedback mechanism of parathyroid hormone-related proteins, and also directly controls chondrocyte proliferation and osteoblast function.^45^

The activation and secretion of Dhh and Ihh are similar to that of Shh. Dhh and Ihh also undergo autocatalytic cleavage to form Dhh-N and Ihh-N, in which the C-terminal structural domain is removed.^46^ This N-terminal structural domain is then modified by N-terminal palmitoylation as well as the cholesterol addition of C-terminal amino acids, resulting in the formation of the mature proteins Dhh-N and Ihh-N.^47^ All three HH ligands share high sequence homology, similar overall fold and are capable of binding to and activating the same signaling pathway receptors. They mainly differ in their N-terminal amino acid sequence; however, their N-terminal sequence similarity may also reach 76–91%.^48^

Over the years, it has also become increasingly clear that Dhh and Ihh have multiple functions in other tissues. For instance, full-length Dhh plays a critical role in regulating vascular endothelial integrity.^49^ The potential roles of Dhh and Ihh in varies organs requires further investigation.

### Receptors and co-receptors

#### Ptch

Ptch, the 12th transmembrane HH receptor, has two homologs, Ptch1 and Ptch2.^50^ The structure of Ptch1 includes the transmembrane structural domain (TMD), which comprises a sterol-sensing structural domain (SSD) and two extracellular structural domains (ECD1 and ECD2) (Fig. 2b).^51^ Various researchers have suggested that Ptch1 acts as a major receptor for HH ligands. Here, modifications of Shh-N may occur in its interaction with Ptch1.^52^ In addition, molecular evidence has shown that Ptch1 is able to recognize the calcium-mediated interface of Shh-N. Moreover, the binding mode of Shh and Ptch1 has been reported to be the formation of the 1 Shh:2 Ptch1 complex (Fig. 2d).^53^ However, in vivo studies have revealed that co-receptors can form a heterotrimeric complex with Ptch1 and HH-N and induce cell proliferation or embryonic development.^29^ Nonetheless, how HH recognizes and inhibits Ptch1 continues to remain controversial.

While the role of Ptch2 is inadequately understood, it compensates the response to Shh in the absence of Ptch1.^54^ Ptch2 may be able to bind HH proteins in different cellular environments in order to regulate the subsequent signaling pathway.^55^

#### Smo

In-depth structural and functional studies have revealed the role of the two variable ligand binding sites, seven-pass α-helical transmembrane bundle (TMD) and extracellular cysteine-rich domain (CRD) of Smo, a G protein-coupled receptor, in regulating Smo activity (Fig. 2c).^56,57^ The TMD and extracellular loops form a ligand-binding region.^58^ CRD at the extracellular amino terminus mediates Smo dimerization. In addition, intracellularly, the carboxy-terminal tail mediates the conformational change of the Smo dimer. In the absence of ligand, the carboxyl terminus is in a closed conformation, obscuring the kinase binding site; in the presence of ligand, the carboxyl terminus ser/thr cluster is highly phosphorylated, forming an open conformation.^59–61^

**Fig. 3:**
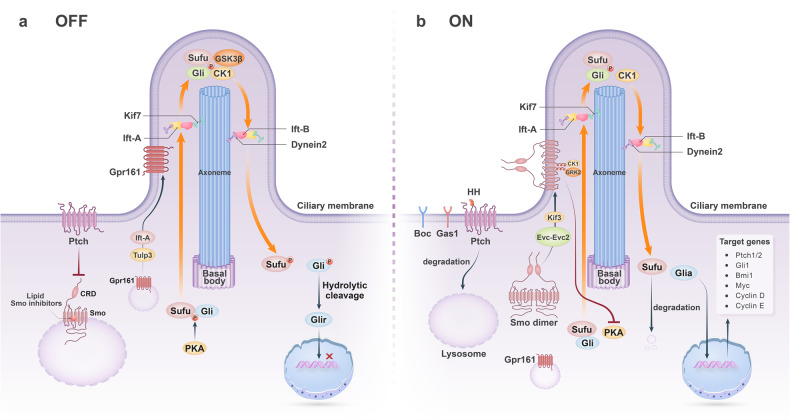
Primary cilia play a key role in HH signaling pathway. Primary cilia are composed of ciliary membrane, axoneme and basal body. The proper functioning of HH signaling relies on the Ift mechanism. Ift-B complex protein and Ift-A complex protein together constitute Ift trains that carry motors with HH signaling components sliding on the axoneme. Kinesin mediates transport from the base to the tip of the cilium, while dynein2 mediates transport from the tip to the base of the cilium. **a** Receptor cells are closed to signal transduction. In the absence of hedgehog ligand, Ptch1 inhibits Smo. Lipid Smo inhibitors bind to the TMD region of Smo, inhibit Smo activity, and constrain Smo to the cytoplasm. Gpr161 localizes to the cilia in the presence of Ift-A and Tulp3. Gpr161 induces PKA activity, and PKA phosphorylates the Sufu/Gli complex. Kinesin Kif7 then transports the complex to the cilia tip. Sufu is further phosphorylated by GSK3β; Gli is further phosphorylated by CK1 and GSK3β. The complex then dissociates and Gli is processed by protein hydrolytic cleavage to Glir, which subsequently enters the nucleus to repress target gene transcription. **b** Receptor cells are open to signal transduction. In the presence of Hedgehog ligand, the inhibition of Smo by Ptch is released and Ptch is transported to the lysosome for degradation. The co-receptors Boc and Gas1 can interact with Ptch1 and Ptch2 to form a receptor complex respectively, thereby promoting or inhibiting signal transduction. The concentration of intracellular lipid Smo inhibitor ligands decreases and Smo forms an activated dimeric form that is transported by Kinesin Kif3 and Evc-Evc2 proteins to the cilia membrane near the basolateral. The Smo carboxyl terminus is then fully activated by phosphorylation of CK1 and GRK2. After Smo activation, Gpr161 returns to the cytoplasm. Meanwhile, activated Smo inhibits PKA activity and is not sufficient to phosphorylate the Sufu/Gli complex. The unphosphorylated complex is transported to the cilia tip by Kinesin Kif7. The unphosphorylated Sufu is degraded by ubiquitination; Gli is phosphorylated by CK1 to form Glia. Glia enters the nucleus to promote the transcription of related target genes, such as Ptch1/2, which acts as a negative regulator of signal transduction, Gli1, which amplifies signals, Bmi1, which encodes a transcriptional repressor, and Myc, Cyclin D, and Cyclin E, which encode cell cycle regulators

In the absence of ligand stimulation, Ptch inhibits Smo to block the cascade reaction. The question of how Ptch inhibits Smo remains relatively mysterious. Most scholars believe that Ptch can modulate the binding of lipid Smo ligands to Smo, which can inhibit Smo activity. These endogenous inhibitory molecules usually refer to sterols or their derivatives (Fig. 2e).^20,62,63^ The inactive Smo is located in the cytoplasm. However, when HH binds to Ptch, the restrictive effect of Ptch on Smo is lifted to initiate downstream signaling pathways.^64,65^ Ptch is transported to lysosomes in the cytoplasm for degradation. Smo activation requires two mechanisms: first, Smo needs to be translocated to primary cilia, a microtubule-based organelle (Fig. 3); second, Smo localized to cilia undergoes carboxy-terminal phosphorylation.^66,67^ A recent study found that cholesterol covalently bound to the D95 residue of the CRD of Smo is required for Smo activation, a process that results from calcium-promoted autocholesterolization of Smo.^68,69^

#### Co-receptors

Brother of Cam-related/downregulated by oncogenes (Boc) and growth arrest-specific 1 (Gas1) have been identified as co-receptors regulating HH signaling pathway activity. The specific heterogeneous complexes formed by their interaction with Ptch1/2 receptors, respectively, can mediate different kinetic Smo depression programs through distinct ligand reception patterns.^70,71^ Notably, Boc sometimes acts as an inhibitor of HH signaling pathway during craniofacial tissues development.^71^ In addition, Hspg can act as co-receptors for neuronal cell development through glypican 5 core protein and 2-O-sulfo-iduronic acid structures.^72^

#### Gli family transcription factors

Upon excitation of HH signaling pathway, Gli transcription factors are activated thereby regulating target gene expression. The vertebrate homologs of this Drosophila Cubitus interruptus transcription factors include Gli1, Gli2, and Gli3 proteins. Gli1 is exclusively a transcriptional activator, whereas Gli2 and Gli3 have dual activity. Although both contain highly similar zinc finger structural domains that can bind to DNA, Gli3 often acts as a transcriptional repressor because the process of protein hydrolysis is most pronounced at Gli3, while Gli2 acts primarily as a transcriptional activator.^73,74^ Through protein hydrolysis, they act as bifunctional transcription factors, changing from the full-length transcriptional activator form (Glia) to the carboxy-terminal truncated transcriptional repressor form (Glir).^11^ GLI-similar (Glis) transcription factors, a subfamily highly similar to Glis, can also undergo nuclear translocation by post-translational modifications in the cytoplasm, and their potential interconnection with Gli1-3 enriches the Gli network.^75–77^

Gli protein activity can be regulated by protein-protein interactions. Sufu, a negative regulator of Gli1-3, was shown to abduct Gli1-3 in the cytoplasm to prevent it from undergoing nuclear translocation.^12,78,79^ In cells, Sufu is phosphorylated by Protein kinase A (PKA) to form a stable phosphorylated form of Sufu, which affects its interaction with Gli.^80^ Sufu and Gli form a complex in the cytoplasm after entering the primary cilia (Fig. 3), resulting in different phosphorylation states of the two proteins and determining Glia or Glir production, depending on the presence or absence of hedgehog ligand stimulation.^81^ In the absence of ligand stimulation, the Sufu/Gli complex is in a closed conformation; after ligand stimulation, Sufu assumes an open conformation in the complex. The different phosphorylation states of both proteins cause conformational changes that lead to dissociation of the Sufu/Gli complex and entry of Gli into the nucleus to regulate target gene expression.^82,83^

#### Hedgehog signaling pathway target genes

HH signaling pathway plays an essential role in cell proliferation and differentiation, tissue development, homeostasis and EMT.^3,16^ Its complex regulatory network influences the transcription of divergent target genes that vary between tissues and cell types (Table 1). In most cells responsive to HH signaling, the common target genes include Ptch1/2 and Gli1.^84^ Moreover, increased expression of the Ptch1/2 and Gli1 genes have been shown indicate an activated HH signaling pathway and could provide negative (Ptch1) and positive (Gli1) regulation of HH signaling with feedback loop mechanisms. Furthermore, in tissue-specific stem cells, HH signaling induces Bmi1, which acts as a transcriptional repressor that regulates the proliferative capacity of cells.^85^ Numerous target genes in this pathway are cell cycle regulators, such as Myc, CyclinD, and CyclinE, as well as development-related regulators, such as Fgf4 and Hhip.^86–89^ In the developing neural tube, this pathway regulates the Nkx6.1, Olig2, Nkx2.2, and FoxA2 genes, conferring ventral neural fates and has Gli-binding sites in the regulatory regions.^90^ In addition, HH signaling pathway promotes the expression of certain genes, including Snzi1, Zeb1, Twist2 and Foxc2, which enhance epithelial mesenchymal transition.^91^ Other target genes include the apoptosis regulator Bcl2, Vegfa, Abcg2, Mycn, Foxm1, Pax6, Pax7, Pax9 and Jag1, as well as members of the Wnt signaling pathway.^92–98^ In summary, HH signaling pathway can regulate the corresponding target gene transcription in different functional states and cell types.

**Table 1:** Hedgehog signaling pathway target genes

Target gene	Full name of the gene	Function of the gene products
Ptch1^84^	Patched 1	Receptor that initiates signaling pathways, negative feedback regulation
Ptch2^84^	Patched 2	Receptor that initiates signaling pathways
Gli1^84^	Glioma-associated oncogene 1	Direct transcriptional activator, positive feedback regulation
Bmi1^85^	Bmi1 proto-oncogene	Transcriptional repressor regulating the proliferative capacity of cells
Myc^86^	Myc proto-oncogene	Cell cycle progression, apoptosis and cellular transformation
CycD and CycE^87^	CyclinD and CyclinE	Cell cycle progression
Fgf4^89^	Fibroblast growth factor 4	Involved in varieties of biological processes including embryonic development, cell growth, morphogenesis, tissue repair and invasion
Hhip^88^	Hedgehog interacting protein	Involved in varieties of developmental processes including anteroposterior patterns of limbs and regulation of left-right asymmetry in embryonic development
Nkx6.1^90^	NK6 homeobox 1	Required for the development of beta cells
Nkx2.2^90^	NK2 homeobox 2	Involved in the morphogenesis of the central nervous system
Olig2^90^	Oligodendrocyte transcription factor 2	Regulator of ventral neuroectodermal progenitor cell fate
FoxA2^90^	Forkhead box A2	Transcriptional activators that regulate the specificity and differentiation of dopaminergic neurons
Snai1^91^	Snail family transcriptional repressor 1	Critical for mesoderm formation in the developing embryo
Zeb1^91^	Zinc finger E-box binding homeobox 1	Associated with posterior polymorphous corneal dystrophy-3 and late-onset Fuchs endothelial corneal dystrophy
Twist2^91^	Twist family bHLH transcription factor 2	Inhibitors of osteoblast maturation, maintaining the pre-osteoblast phenotype of cells during osteoblast development
Foxc2^91^	Forkhead box C2	Involved in the development of mesenchymal tissues
Pax6, Pax7 and Pax9^97^	Paired box 6, 7 and 9	Involved in the development of neural tissues

## Hedgehog signaling and the primary cilium

Unlike other signaling pathways, the normal maintenance of the vertebrate canonical HH signaling pathway is highly dependent on primary cilia. Primary cilia recruit extracellular signaling components that link the extracellular environment, where signals are complex and diverse, to the intracellular environment.^13,99^ This section will briefly describe the structural features of primary cilia and the way in which the key components of HH signaling pathway act in primary cilia.

### Brief introduction of primary cilia

The primary cilium is a specialized structure located on the cell surface.^100^ The axoneme of primary cilia includes 9 microtubule doublets in the outer ring, while lacking the central 2 microtubule singlets (known as 9 + 0 axoneme). Primary cilia are anchored to the cell by basal body, which is specialized from the mother centriole and contains a number of proteins for ciliogenesis and assembly.^101,102^ Between the cilia and basal body is the ciliary transition zone, which regulates protein transport within the cytoplasm and cilia.^103^

The transport of protein molecules within primary cilia to the cytoplasm is achieved through the Ift mechanism.^14^ Ift is driven by two molecular motors, involving cytoplasmic dynein2, which mediates transport from tip to base, and the heterotrimeric kinesin-2 complex comprised of Kif3a, Kif3b, and Kap3, which is responsible for transport from base to tip.^15,104,105^ Mutations in the gene encoding the heavy chain of cytoplasmic dynein2 cause axoneme swelling because Ift-related proteins are trapped in cilia due to disruption of retrograde transport.^14^ Gli cooperates with Kif3a and Kap3, where the interaction of Kap3 restricts Gli transcriptional activity.^106^ Ift-B complex protein and Ift-A complex protein together constitute Ift trains that bridge kinesin motors with HH signaling molecules.^107^ Ift trains slide back and forth along the axoneme and are responsible for cilia structure assembly and balancing. Periodic binding of Ift-A, Ift-B and dynein2 can balance the protein entry and exit of cilia, effectively preventing the accumulation of material.^108,109^ Huang et al. showed that wimple mutants disrupting Ift172, an Ift-B protein, lacked the normal primary cilia structure, which resulted in the inability of the receiving cells to respond to Shh.^110^

Changes in the structure and function of primary ciliary protein fractions can have an impact on HH signaling pathway. Defective RNAi of Kinesin Kif14 disrupts the function of distal appendage proteins Sclt1 and Fbf1, which results in a defective basal body that fails to activate HH signaling pathway.^111^ Disruption of most primary ciliary proteins decreases signaling activity, but some proteins are altered in different ways that enhance pathway activity; thus, changes in ciliary structure have a bidirectional effect on the HH signaling cascade response, with subsequent effects on cell fate.^15,112^

### Hedgehog signaling functions through primary cilia

#### Smo and primary cilia

The ciliary membrane contains a specific receptor for HH signaling pathway, Ptch, which is essential for receiving the initial signal from the outside.^113,114^ In the absence of Hedgehog ligand, Ptch induces a closed conformation of Smo by controlling the TMD binding of small lipid Smo ligands to Smo, allowing Smo to remain in the cytoplasm (Fig. 3a).^63,115^ In the presence of Hedgehog ligand, Ptch derepresses Smo and Smo activates. Activation of Smo involves its localization to the ciliary membrane and carboxy-terminal phosphorylation.^59,116–118^ First, the concentration of small lipid Smo inhibitors in the cytoplasm decreases and Smo transforms into an open conformation and is transported to the ciliary membrane by the action of the kinesin Kif3.^119,120^ Besides, after reaching the cilia, the activation of Smo depends on the concentration of Hedgehog ligands. When there is no sufficient ligand to stimulate the signal, the small lipid Smo inhibitor rapidly binds to the TMD again and the Smo changes back to a closed conformation and returns to the cytoplasm.^121^ When stimulated by sufficient Hedgehog ligands, the Smo carboxyl terminus is fully activated by casein kinase 1 (CK1) and G protein-coupled receptor kinase 2 (GRK2) phosphorylation (Fig. 3b).^60,61^ It can be seen that Smo acts in a Gαi - coupled Gpcr manner, which is highly sensitive to changes in Hedgehog ligand concentration.

#### Gli and primary cilia

In the absence of Hedgehog ligands, Gpr161, a G protein-coupled receptor, enters the cilia and localizes to the ciliary membrane via Ift-A and Tulp3, a member protein of the vertebrate tubby-like family.^122,123^ Then, in the presence of adenylate cyclase 5/6 (Ac5/Ac6), cAMP increases and activates PKA, which phosphorylates both proteins of the Sufu/Gli complex.^124,125^ Gli is further phosphorylated by CK1 and Glycogen Synthase Kinase-3 (GSK3β) and forms Glir by proteolytic cleavage (Fig. 3a).^123,126,127^

In the presence of Hedgehog ligands, Kinesin Kif7 dephosphorylates and then aggregates at the tip of primary cilia, in parallel with the return of Gpr161 to the cytoplasm.^128,129^ Gαi inhibits Ac5/Ac6 and reduces PKA activity after intraciliogenic activation.^130–132^ In addition, Evc-Evc2 proteins, which localize active Smo at the base of the cilia, also inhibit PKA activity.^133^ The Sufu/Gli complex, which is not phosphorylated by PKA, is transported to the cilia tip by Kinesin Kif7 and accumulates there.^134^ Within the cilia, unphosphorylated Sufu is degraded by Scf-mediated ubiquitination; however, Gli is phosphorylated by CK1 to form Glia.^135^ Then the complex dissociates and Glia enters the nucleus to mediate the transcription of target genes (Fig. 3b).^117,121^

Because CK1 and GSK3β, which are required for the phosphorylation process of the Sufu/Gli complex, are present in primary cilia, primary cilia are necessary to determine the form of Gli.^136^ Furthermore, the cilium is a local compartment relative to the cytoplasm, therefore small changes in PKA activity can be detected in the little space, which determines changes in Glia versus Glir.^125,137^

## Hedgehog signaling in homeostasis

Well known for its crucial role in regulating the embryonic and postnatal development of vertebrate, HH signaling pathway has been proven to orchestrate the specific formation and morphogenesis of an organism.^5,99,138,139^ Meanwhile, HH signaling pathway has been demonstrated to express continually in many adult mammalian tissues and organs, especially in the epithelial and mesenchymal cells, maintaining the homeostasis of adult tissues and organs via regulating a great variety of quiescent stem cell populations as well as the epithelial-mesenchymal interactions.^99,140,141^ This part of review outlines recent discoveries on the crucial roles of HH signaling pathway in the homeostasis of multiple tissues and organs possessing representative characteristics.

### HH signaling pathway maintains the homeostasis in the mesenchyme

HH signaling pathway is one of the major signaling pathways regulating osteogenesis and post-embryonic long bone homeostasis with postnatal expression in the mesenchyme.^142,143^ Yang et al. found that silencing Ift80 impairs cilia formation and reduces HH signaling pathway expression in bone.^144^ Ift80 increases the expression of osteoblast markers by regulating HH/Gli pathway, demonstrating that HH signaling pathway plays a remarkable role in maintaining osteoblast differentiation and mineralization. Ihh upregulates mesenchymal stem cells (MSCs) and osteoblast markers. Tissue engineering experiments utilizing Ihh-MSCs-scaffold complex has shown increased bone repair capacity.^145^ Shh is a potential signaling molecule that regulates osteoblast differentiation. Armstrong et al. have shown that Shh in zebrafish can upregulate the expression of Sp7 in osteoblast lineage cells and increase the proliferation of osteoblasts.^146^

The calvarial bone has become an emerging model for studying the function of HH signaling pathway in bone homeostasis. Regarded as the primary niches for osteogenesis, craniofacial sutures contain MSCs.^140,147,148^ Zhao et al. revealed that cells harboring MSCs characteristics were Gli1^+^. These MSCs play a significant role in regulating calvarial bone formation and injury repair.^147^ Using diphtheria toxin to disentangle Gli1^+^ cells led to premature fusion within the cranial sutures, resulting in craniosynostosis, indicating the vital role of HH signaling pathway in controlling the suture homeostasis and calvarial bone patterning and repair.^147,149^ Moreover, the interplay between BMP and Ihh signaling has been identified in maintaining suture homeostasis via interaction among MSCs, osteoprogenitors, and osteoclasts during calvarial bone formation and repair.^150^

Skeletal muscle has the ability to repair damage due to the presence of muscle stem cells, namely, myosatellite cells.^151^ Myosatellite cells normally maintain a quiescent condition and give rise to myogenic cells and reform the myofibers of the muscle in response to injury.^152^ Study showed that muscle injury could stimulate Shh expression. When Shh signaling was inhibited, the expression of Myf5 and MyoD was impaired, accompanied by reduced number of activated myosatocytes.^153^ Similarly, Koleva et al. showed that Shh could promote the proliferation of myosatellite cells and myotube fusion.^154^ Subsequently, Elia et al. found that Shh can promote the differentiation of chicken primary myoblasts, and this effect can be inhibited by cyclopamine, an inhibitor of Shh signaling.^155^ It has also been reported that Shh promotes the expression of Pax7, Myf5, MyoD, MyoG, and MyHC in mouse myoblasts, and the promotion of these genes by Igf-1 depends on HH signaling pathway.^156,157^ Further study by Voronova et al. showed that Gli2 formed a protein complex with MyoD and Mef2c to enhance the transcription factor activity of MyoD.^158^ MyoD regulates the activity of HH signaling pathway, which allows Gli2, Mef2c, and MyoD to form a reciprocally regulated recycling network. Devakanmalai et al. reported that HH signal affects muscle cell differentiation by activating MyoD and Myf5, and then regulating Mef2c. These studies indicate that HH signaling pathway is essential for postnatal muscle homeostasis.^159^

HH signaling pathway may also participate in smooth muscle regeneration and differentiation. Kramann et al. found that adventitial MSC‐like Gli1^+^ cells were progenitors of vascular smooth muscle cell and expressed CD34, Sca1, and Pdgfrβ, possessing tri-lineage differentiation capacity towards osteoblasts, adipocytes, and chondrocytes.^160^

### HH signaling pathway maintains the homeostasis in epithelial tissue

During the hair cycle, Shh signaling pathway is essential for maintaining hair follicle stem cell populations and regulating the development of hair follicles and sebaceous glands, meanwhile regulating epithelial-mesenchymal interactions.^161,162^ Mammalian epidermal metabolism is maintained by the continuous proliferation of epidermal stem cells.^163^ Adolphe C et al. found that Ihh overexpression did not result in any significant epidermal morphogenetic phenotype, while overexpression of Dhh was indistinguishable from Shh. The phenotypes were resulted from dysregulation of stem cell activity, including hyperplasia of epidermal progenitors and almost complete loss of epidermal tissue renewal capacity, indicating that HH activity is the vital factor to maintain homeostasis of epidermal stem cells.^164^ Zhou et al. showed that Shh, its receptors Ptch1, Smo and its downstream transcription factor Gli1 were detected in the basal layer of fetal epidermis and in newly sorted human putative epidermal stem cells (HPESCs).^165^ HPESCs treated with medium containing Shh-N exhibited enhanced cell proliferation. In contrast, cyclopamine inhibits Shh and thus prevents proliferation. Similarly, the mitogenic effect of epidermal growth factor on HPESCs can be eliminated by cyclopamine. In addition, the expression of Bmp-4, a potential downstream effector of Shh signaling, can increase HPESCs proliferation.^165^ Shh signaling in mammalian skin controls hair follicle epithelial cell growth and morphogenesis by regulating Gli transcription factors.^166^ Shh expression and the ability of skin cells to respond to Shh signaling pathways are spatially and temporally regulated during the hair cycle.^167^ Knockout of Shh-dependent Sox9 in the skin results in the appearance of external hair, with severe proliferation defects and difficulty in forming a stem cell microenvironment.^168^ Additional studies have shown that the demand for substrate formation by β-catenin occurs downstream of Tabby/Downless and upstream of Bmp and Shh.^169^

Intestinal epithelial homeostasis is regulated by a strictly controlled balance between intestinal stem cell (ISC) proliferation and differentiation.^170^ *Villin-Cre;Ihh*^*flox/flox*^ mutant mice show a significantly reduced number of villi and reduced cell proliferation in the stem cell compartment, suggesting that Ihh is essential for ISC regeneration and differentiation.^171^ Specifically, deletion of Ihh in intestinal epithelial is accompanied by an increase in epithelial Wnt signaling pathway, while the activation of the Wnt pathway is a common cause of intestinal tumorigenesis.^172,173^ HH might have a tolerogenic influence as a regulator of inflammation.^174^ Lees et al. showed the reduced expression of HH signaling pathway in individuals with inflammatory bowel disease and ulcerative colitis.^175^ Dop et al. conditionally deleted Ihh in adult mice and found an inflammatory response accompanied crypt changes, resulting in influx of macrophages and fibroblasts into the villus core.^176^ Recent studies suggest that more than one target cell population (e.g., macrophages, dendritic cells) is relevant to HH-modulated inflammation.^177,178^

### HH signaling pathway maintains the homeostasis via regulating epithelial-mesenchymal interaction

Epithelial-mesenchymal interactions coordinated by HH actively maintain homeostasis and regulates repair and regeneration in lung.^179^ Tien et al. found proliferative expansion of the adjacent lung mesenchyme when specifically deleting Shh in the murine lung.^180^ Reduced expression of HH signaling pathway is initially detected during the acute phase of epithelial injury as the mesenchyme proliferates in response, whereas being upregulated to the baseline while the homeostasis is restored. However, Liu et al. demonstrated the upregulated HH signaling pathway in lung fibrosis or airway injury, supported by a proliferation in stromal Gli1^+^ cells in adult mice.^181^ Moreover, when confronted with an airway injury, Shh overexpression enhances collagen deposition and lung fibrosis.

Both epithelial and mesenchymal compartments of the rodent incisors continue to grow and regenerate throughout the lifespan of animals and requires constant repair. It has been identified that during the growth of the mouse incisor, Shh plays a significant role in maintaining the ability of stem cells to expand the ameloblast lineage in the incisor epithelium, indicating that Shh regulates the homeostasis of mouse incisor enamel, the epithelial compartment of incisors.^182^ Meanwhile, Shh signaling pathway also plays an important role in the homeostasis and regeneration of dentin, the mesenchymal compartment of incisors. Zhao et al. identified that Gli1^+^ MSCs were localized around arteries and the accompanying nerves, while the HH ligand that maintains the Gli1^+^ MSC population was secreted by nerves in the neighboring neurovascular bundle (NVB).^183^ In addition, Gli1^+^ cells were also indicated to contribute to incisor injury repair by generating reparative dentin.

Unlike the rodent incisor, the rodent molar does not possess the property of continuous growth, and the majority of Gli1^+^ cells were distributed within the mesenchyme, mainly in the periodontal ligament near the molar apical. These cells were activated and significantly contributed to the bone remodeling when imposed on the orthodontic forces.^184^

## Hedgehog signaling in cancer

**Fig. 4:**
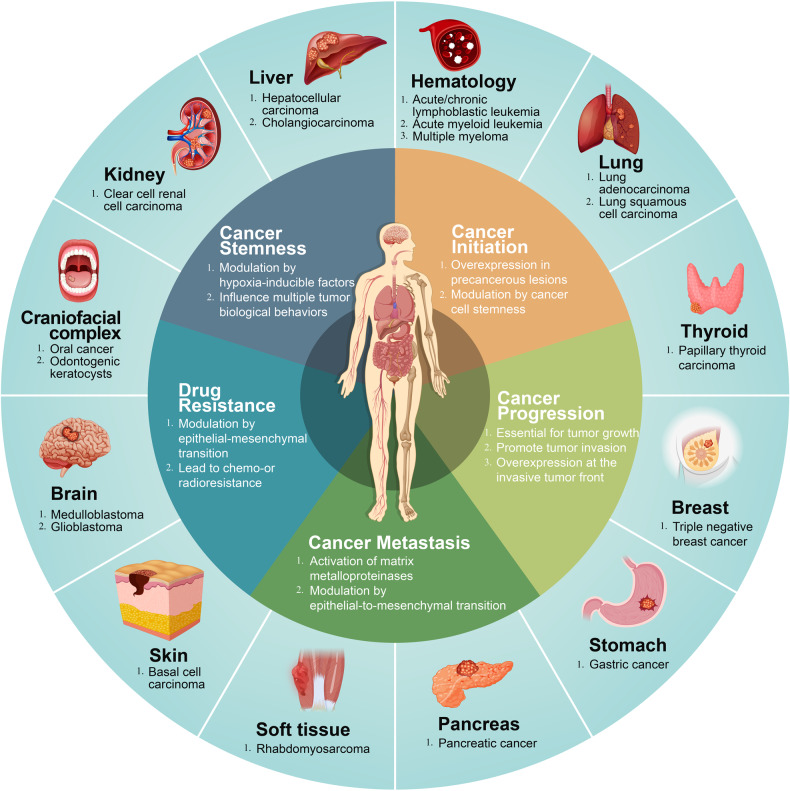
Summary of types of cancer caused by dysregulation of HH signaling pathway. The inner circle present five aspects of tumor biological behavior which are influenced by HH signaling pathway. The outer circle shows the representative cancers discussed in the review. The online resources in the picture were obtained from the website: www.699pic.com and www.vecteezy.com

Cancer is one of the major causes of death in the world. Almost ten million cancer deaths were expected in 2020.^185^ Cancers are highly burdensome diseases, which have negative physical and mental effects on patients, as well as serious economic consequences. HH signaling pathway has been documented to be responsible for tumor initiation and premalignant lesion as well as tumor progression leading to a greater tumor size and more invasive behavior.^186–188^ Tumor metastasis and the resistance to anti-cancer therapy (radio and chemo-resistance) were the ultimate challenges to fight cancer as a life-threatening disease.^189,190^ Also, cancer stem cell (CSC) with the self-renewal ability not only has a close relationship with multiple tumor properties mentioned above such as tumor initiation, development, metastasis, and tumor recurrence.^191^ Therefore, we mainly discussed the decisive role of HH signaling pathway in five aspects including cancer initiation, cancer progression, metastasis, resistance to anti-cancer therapy, and cancer stemness in different organs in this section (Fig. 4).

Cancer cells in conjunction with the surrounding oncogenic stromal components form the tumor. Microvascular components, fibroblasts, endothelial cells and other noncellular elements in the tumor stroma determine the multiple behaviors of tumors, such as tumor initiation, progression, and resistance to anticancer therapy.^192^ The interplay of HH signaling pathway between cancer cells and the stroma component may be overall described using the following models. Mutations of HH signaling pathway components, such as Ptch and Smo, drive ligand-independent HH signaling pathway activation, which can lead to systemic disease and manifest with a variety of symptoms, such as Gorlin syndrome. Cancer cells have also been shown to secrete HH ligands in an autocrine manner in cancer progression.^193,194^ In addition, ligand-dependent activation of the HH pathway in tumors have been shown to adopt a paracrine or a reverse-paracrine manner.^195,196^

A plethora of studies have supported the presence of a dynamic interaction via mutual transmission of HH signaling molecules between tumor cells and stromal cells. Coexistence of such HH ligand interactions in different types of tumors has also been shown to be conclusive. The association of HH signaling pathways with different cancers, including changes in related molecules, secretory mechanisms, and tumor characteristics were summarized (Table 2).

**Table 2:** Changes of HH signaling pathway in cancer

Cancer	Mechanism	HH signaling pathway-related molecules	Biological behavior influenced by HH signaling pathway
Craniofacial Complex			
Oral squamous cell carcinoma^198,199^	ligand-dependent	Ptch1 (+), Shh (+), Smo (+), Gli1 (+)	Initiation (+), Progression (+), Metastasis (+)
Odontogenic karatocytes^211^	ligand-independent	Ptch1 (+), Smo (+)	Progression (+)
Brain			
Medulloblastoma^226,230,232^	ligand-independent	Ptch1 (+), Shh (-)	Stemness (+), Progression (+), Resistance (+)
Glioma^237,510^	ligand-dependent	Shh (+), Gli2 (-)	Progression (+), Metastasis (+)
Renal			
Renal cell carcinoma^249,254,255,511^	ligand-dependent	Shh (+), Smo (+), Gli1 (+), Gli2 (+)	Stemness (+), Progression (+), Resistance (+)
Prostate			
Prostate cancer^259,263,512^	ligand-dependent	Shh (+), Smo (+)	Stemness (+), Progression (+), Resistance (+)
Liver			
Hepatocellular carcinoma^266,267,271,274^	ligand-dependent	Ptch1 (+), Smo (+), Gli1 (+)	Progression (+), Metastasis (+), Resistance (+)
Cholangiocarcinoma^276,282,513^	ligand-dependent	Gli1 (+), Gli2 (+)	Stemness (+), Progression (+), Metastasis (+), Drug resistance (+)
Colon			
Colorectal cancer^284,287^	ligand-dependent	Ihh (-), Shh (+),	Progression (+), Metastasis (+), Resistance (+)
Pancreas			
Pancreatic cancer^289–291,294^	ligand-dependent	Shh (+), Smo (+), Gli1 (+)	Initiation (+), Progression (+), Metastasis (+)
Lung			
Lung cancer^299,304,305,514^	ligand-dependent	Shh (+), Gli (+)	Progression (+), Stemness (+), Metastasis (+), Resistance (+)
Blood			
Hematological malignancy^308,311,515^	ligand-dependent	Smo (+), Dhh (+), Gli1 (+)	Radiation resistance (+), Stemness (+)
Thyroid			
Thyroid cancers^320,324,516^	ligand-dependent	Shh (+), Ptch (+), Smo (+), Gli (+)	Stemness (+), Progression (+), Resistance (+)
Skin			
Basel cell carcinoma^327,329,335^	ligand-independent	Shh (+), Ptch1 (+), Gli1 (+)	Progression (+), Resistance (+)
Breast			
Triple negative breast cancer^341,342,345,346^	ligand-dependent	Smo (+), Gli1 (+), Shh (+)	Progression (+), Metastasis (+), Resistance (+), Stemness (+)
Muscle			
Rhabdomyosarcoma^351,357^	ligand-dependent	Gli1(+), Ptch1 (+)	Progression (+), Stemness (+), Drug resistance (+)
Stomach			
Gastric cancer^364–367^	ligand-independent	Shh (+), Gli1(+)	Initiation (+), Progression (+), Drug resistance (+), Stemness (+)

### Cancers in craniofacial complex

Squamous cell carcinomas account for more than 90% of oral malignant tumors and often begin with precancerous lesions.^197^ Shh expressed in oral epithelial dysplasia and carcinoma in situ.^198^ Also, Shh, Gli2, Smo, and Ptch are highly expressed in precancerous lesions of oral mucosa.^199^ Higher expression levels of Ptch1, Smo, and Gli1 were found in oral squamous cell carcinoma (OSCC) cases when compared to nonneoplastic oral mucosa. Recent study demonstrated that Gli1 was present only in the nuclei of cancer-associated fibroblasts in OSCC, indicating that HH signaling is active in the progression of OSCC.^200^ Furthermore, endophytic-type parenchyma of OSCC possessed a stronger expression of Shh and Ptch1 than exophytic-type parenchyma of OSCC.^201^

HH signaling molecules are considered to be prognostic indicators in OSCC as overexpression of related proteins can be indicative to tumor size, metastatic potential, tumor recurrence and even to shorter overall survival.^202,203^ HH signaling regulates cell proliferation in OSCC.^204^ Several studies have investigated the molecular mechanisms on the invasion behavior of human OSCC. Ptch1 and Gli expression were found in the microvascular cells in the invasive front of OSCC.^205^ Takabatake et al. reported that Ptch and CD31 double-positive blood vessels in the OSCC stroma might affect tumor angiogenesis in OSCC.^201^ Also, Shh overexpression is correlated with cancer stem cell markers CD133 and Sox2 in OSCC specimens.^202^ Shh might induce OSCC progression and invasion through prolonged half-life of activated leukocyte cell adhesion molecule (ALCAM) which is a transmembrane glycoprotein mediating cell adhesion and multiple other function of cancer cell.^206^ An increased MMP-9 and downregulated epithelial cadherin (E-cadherin) expression caused by Shh signaling in OSCC might be responsible for the invasiveness and metastatic potential.^207^

Odontogenic keratocysts (OKCs) accounts for 3.3–17.4% of all jaw cysts, of which neoplastic lesions often involve mandibles with a high clinical risk of recurrence.^208,209^ Gorlin syndrome (GS) is a multisystem genetic disease caused by gain of function mutation in HH signaling pathway and manifests as multiple OKCs and basal cell carcinomas (BCCs).^209,210^ The most common gene mutation for GS is Ptch1 while Ptch2, Smo and Sufu are also rare causative genes for GS.^211^ Although multiple OKCs is a major symptom of GS, even patients with sporadic OKCs were suspected with an underestimated Ptch1 mutation rate.^212^ Stojanov et al. demonstrated Ptch1 inactivating mutations accounts for 93% of sporadic OKCs.^213^ Yu et al. indicated that mutations in the intracellular loop of Ptch1 might regulate Cyclin B1 in NBCCS-associated OKCs in a manner of noncanonical HH signaling pathway.^214^ Ptch1 mutation leads to ligand-independent activation of Smo and subsequently upregulates HH signaling pathway target genes transcription.

Many studies concluded that the aberrant HH signaling pathway is active in the development of OKCs.^215,216^ Grachtchouk et al. uncovered that elevated expression of HH target genes is detected in lower cell layers of cyst wall of human OKCs.^217^ Besides, OKCs were found to express lower levels of Sufu gene and higher level of Smo, Ptch1, Cyclin D1 and Bcl2.^218^ Syndromic OKCs were found to have a higher expression of HH signaling pathway protein than the sporadic OKCs.^219^ Higher Smo expression was considered to be a risk factor of recurrence of OKCs.^220^

### Brain tumor

Brain tumor and other neoplasm in nervous system account for 1.6% new cases worldwide in 2020 and lead to more than 251,329 deaths.^185^ Two types of malignant primary brain tumors including medulloblastoma (MB) and glioma were in close relationship with HH signaling pathway.^221^ MB is a common aggressive malignant tumor in brain during childhood.^222^ There are four subtypes of MB based on research on their molecular mechanism and clinical characteristics: Wnt-MB, Shh-MB, group 3 MB and group 4 MB.^223^ The novel feature of Shh-MB is constitutive activation of the Shh signaling pathway.

HH signaling stimulates the proliferation of cerebellar granule neuron precursors (CGNPs) during cerebellar development, which lead to MB formation.^224^ Researches have indicated that germline and somatic mutation of HH signaling pathway related components leads to MB and such mutation varies in different ages.^225^ However, Ptch1 represents the most common oncogenic mutations in Shh-MB.^226^ Nearly half of patients with Shh-MB harbored Ptch1 (45%) alterations while Smo (14%) and Sufu (8%) alterations were also detected.^227^ Mouse model with conditional deletion of Ptch1 in CGNPs caused MB formation, suggesting that CGNPs play a central role in the origin of Shh-MB.^228,229^ Astrocytes in the cerebellum perform important functions that support granule cell proliferation and migration in the physiological state while MB-associated astrocytes can secrete Shh ligand that helps maintain proliferation tumor cell in a Ptch1-independent manner.^230^

HH signaling pathway has effect on multiple targets including Mycn, Snail1, Cyclin D1, Sox2, Sox9 and further influence cancer behavior as tumor growth and tumor cell proliferation during MB tumorigenesis.^231^ Quiescent cells with a therapy-resistant characteristic can serve as a reservoir for relapse. Sox2, a neural stem cell marker, is recognized as a poor prognosis marker in human Shh-MB. In a mouse model of Shh-MB, cells expressing Sox2 accounts for a proportion of less than 5% of the total tumor cells but might serve as a reservoir for relapse.^232^

Glioblastoma multiforme (GBM) is a common malignant brain tumor and account for 23% of all gliomas.^233^ Given to characteristics of high invasiveness and drug resistance, GBM is a neoplasm with a dismal prognosis and an effective practical therapeutic approach remains under investigation.^234^ Researchers have founded that HH signaling pathway is activated in human glioma cell lines, but not in cultured human astrocytes.^235^ HH/Gli1 pathway affects the growth of glioma and glioma stem cells are thought to be generated by the population of Gli positive neural stem cells. Researchers reported that Gli1 and Gli2 expression is associated with grades III and IV gliomas with less survival.^236^ The overall survival of patients with glioblastoma is decreased by upregulation of Shh based on data from the Cancer Genome Atlas glioblastoma.^237^

Cell migration and proliferation in human GBM cell lines are associated with expression of connexin 43, an integral membrane protein within gap junctions, upon HH signaling pathway modulation.^238^ Chang et al. enhances HH signaling pathway in GBM cells by recombinant human Shh N-terminal peptide, which increases the production of MMPs to promote cell migration and invasion through the PI3K/AKT pathway.^239^ Overexpression of fms related tyrosine kinase 1 (FLT1) in GBM cells was related to the invasion and migration of tumor cells through HH signaling pathway.^240^ Although, Shahi et al. reported a fainter expression of HH signaling pathway component in neuroblastoma (NB) than MB or glioblastoma.^241^ Studies also reported that HH signaling pathway determines multiple biological behavior of neuroblastoma,^242–244^ while several researchers proposed theories of a tumor-suppressive functions of HH signaling pathway in NB.^243,245^

### Renal cancer

Renal cell carcinoma (RCC) is the most common renal tumor, accounting for 80–85% of all renal cancers, as clear cell renal cell carcinoma (CRCC) is responsible for the vast majority of RCC.^246,247^ Dormoy et al. reported HH signaling pathway components are expressed in human CRCC tumor samples, which participates in cell proliferation of CRCC cell lines and related tumor growth. Such reactivation of HH signaling pathway leads to regulation downstream gene transcription of Cyclin D1, PAX2, VEGF, and TGF-β.^248^ The mRNA levels of Shh, Smo and Gli1 were higher in CRCC tissue compared with control kidney tissue in different degrees.^249^ Gli3, Ptch1, Dhh, and Shh were highly expressed in in higher grade tumor as Dhh expression can be recognized as an independent predictor of CRCC survival.^250^ Multiple studies show antitumor potential in human RCC by targeting HH signaling pathway.^251,252^

HH signaling pathway influence aggressiveness of RCC. Recombinant Shh protein enhanced cell proliferation and suppressed the expression of E-cadherin in RCC cell lines, suggesting the essential role of EMT modulating by HH signaling pathway in RCC.^253^ HH signaling pathway may interact with hypoxia-inducible factor 2α modulating the radiosensitivity of RCC.^254^ Furukawa et al. suggested that Gli2 may be related to the underlying mechanism of drug resistance associated with tyrosine kinase inhibitor (TKI) inhibitors sunitinib in CRCC.^255^

### Prostate cancer

Prostate cancer (PCa) is the second most common cancer in male accounting for 14.1% new cases worldwide.^185^ Paracrine signaling of HH signaling pathway is crucial to prostate development.^234^ PCa originates from prostatic epithelia and epithelial-mesenchymal interaction plays an important role in PCa progression and metastasis.^256,257^ Wilkinson et al. has shown the co-localization of Smo with primary cilia in prostatic fibroblasts and confirmed the activated HH signaling pathway in prostatic tumor microenvironment.^258^ Also, Shh expression level was correlated with cell proliferation in PCa. HH signaling pathway blockade by cycloplamine, a selective inhibitor of Smo, significantly reduced the proliferation of PCa cell lines.^259^ Enhanced expression of HH signaling pathway components was found in the cancer tissue than in the normal prostatic epithelial tissue, which was correlated with higher Gleason score and worse prognosis.^260,261^ In animal models, overexpression of Hedgehog protein persistently in mutant mice accelerated the progression of prostatic intraepithelial neoplasia that led to PCa. Hyperplastic basal cells might be the true cellular origin of primary PCa. HH signaling pathway plays important roles in transforming normal prostate basal/stem cells into PCa stem cells.^262^ In addition, inhibition of Shh signaling pathway showed potential for prevention of drug resistance such as zoledronic acid.^263^

### Liver tumor

HH signaling pathway is considered to play an essential role in liver organogenesis and hepatic tissue repair.^264,265^ Importantly, HH signaling pathway is influential to multiple biological behaviors of liver tumor including hepatocellular carcinoma (HCC) and Cholangiocarcinoma (CCC).

The most common malignant liver tumor is HCC.^185^ Ptch1 was found overexpressed in HCC tissue compared with the surrounding non-neoplastic liver tissue. Moreover, increased expression of Smo and Gli1 was directly correlated to a large tumor size of HCC.^266,267^ Ptch1 and Gli1 can be potential biomarkers for the recurrence of HCC and cumulative survival of HCC patients.^268^ MMPs promote tumor metastasis by influencing extracellular matrix remodeling. Shh signaling pathway induces MMP-2 and MMP-9 production to promote invasiveness through FAK/AKT signaling in HCC.^269^ HCC’s invasive behavior was attenuated by treatment with a Smo inhibitor which partially suppressed the expression of MMPs and Gli1/2.^270^ In addition, target genes of HH signaling pathway such as EMT transcription factors were found overexpressed in poorly differentiated hepatoma cells in HCC. Activation of HH signaling pathway and related EMT factors might be responsible for the invasiveness and chemoresistance in poorly differentiated hepatoma cells.^271,272^

A specific Gli inhibitor GANT-61 significantly suppressed HH signaling to reverse sorafenib resistance in CD44-positive HCC, suggesting HH signaling pathway can influence HCC drug resistance.^273^ Further experiments by Zhou et al. confirmed that Gli1/2 binds to the promoter of transporter associated with antigen processing 1 (TAP1) gene, indicating that TAP1 is one of target genes of HH signaling pathway in HCC cell lines. RNAi targeting Gli or TAP1 can alleviating drug resistance.^274^

CCC is a malignant tumor derived from biliary epithelial cells. According to different anatomical positions, it can be classified into intrahepatic, perihilar, and distal CCC, among which perihilar CCC represents about 50% of all cases.^275^ Riedlinger et al. revealed a significant activation of HH signaling pathway in CCC samples and cell proliferation of CCC cells was significantly reduced by inhibiting HH signaling pathway.^276^ The potential effectiveness was also confirmed in a CCC xenograft model that treatment with a combined therapy with BMS-833923 and gemcitabine inhibited tumor growth. Furthermore, HH signaling pathway components such as Gli1 and Gli2, are reliable prognostic factors for CCC.^277,278^

Hypoxia influences multiple tumor biological behavior. Hypoxia inducible factor-1 (HIF-1) promotes cancer stemness and invasive behavior of CCC by modulating Shh, Smo and Gli1.^279,280^ HH signaling pathway protein might promote the interaction between cancer cell and stromal cell and eventually further promotes cancer progression. Coculture of CCC cells with hepatic stellate cell line Lx-2 increased cancer cell migration and invasion.^281^ CCC cells in conventional and hypoxic conditions have been observed to promote tumor-associated macrophage polarization and TGF-β1 secretion via paracrine Shh ligands, thereby promoting CCC cell growth, metastasis and endoplasmic reticulum homeostasis via TGF-β1.^282^

### Colorectal cancers

Among all malignant tumors, colorectal cancer (CRC) ranks third in the world.^185^ HH signaling pathway in the gastrointestinal tract relies mainly on paracrine secretion for completion. In mouse models, the overexpression of Shh ligands provided advantages in growth to tumor cells by activating HH signaling pathway in the surrounding stroma.^196^ Further studies have shown that Shh ligands are upregulated in CRC in order to activate the stromal HH signaling pathway, while stromal downstream genes, such as Gli1 and Hhip expression, are decreased.^283^ This could explain the negative results and Shh ligand overexpression that were evident in clinical trials involving vismodegib for CRC. Several studies have indicated that the downregulation of Ihh can be observed as an early event in the formation of CRC.^284,285^ Ihh is mainly expressed in the stromal tissue of the colon, and its high expression level has been described to inhibit the development of CRC.^283^ In a mutant mouse model, colorectal epithelial cells were shown to secrete Ihh to maintain the intestinal stromal phenotype, which was essential to adenoma development, suggesting that Ihh could influence colorectal malignancies in a variety of manners.^286^ Hypoxic environments can induce the binding of HIF-1α produced by tumor cells and TGF-β2 secreted by cancer-associated fibroblasts to activate Gli2 expression in CRC cells, thus leading to elevated resistance of CRC cells to chemotherapy.^287^

### Pancreatic cancer

Overall 5-year survival of pancreatic cancer is below 10%.^288^ Shh is aberrantly expressed in pancreatic intraepithelial neoplasia and HH signaling remains active in cell lines established from primary and metastatic pancreatic adenocarcinomas.^289^ High expression of Shh and Gli1 was an independent prognostic factor for worse survival of patients with pancreatic ductal adenocarcinoma (PDAC).^290^ Moreover, pancreatic cancer-associated fibroblasts show overexpression of Smo that lead to activation of HH signaling pathway.^291^

HH signaling pathway influences multiple tumor cell behavior in pancreatic cancer through regulating Gli1 expression. Gli1 expression is essential for PDAC cell survival by facilitating the migration and invasion of cells by promoting EMT. S100A4 is an EMT indicator protein and might be an essential target gene mediated by Gli1 in pancreatic cancer.^292,293^ However, Lee et al. proposed that stromal response to HH signaling pathway is protective against PDAC and that HH signaling pathway activated by SAG21k can decelerate tumorigenesis.^294^ Perineural invasion is an important characteristic of pancreatic cancer with an incidence of 70–100%. Cancer cells invade into peripheral nerves in pancreatic tissue and are considered to be associated with poor tumor prognosis and cancer pain.^295,296^ Moreover, overexpression of Shh activates HH signaling pathway in pancreatic stellate cells in the tumor stroma which is not only essential for tumor growth but also responsible for the nerve invasion in cancer.^297^

### Lung cancer

Similar to many cancers, HH signaling pathway is also important for the prediction of lung cancer prognosis.^298^ Inhibition of Shh signaling by cycloplamine induces a significant decrease in the proliferation of Non-Small-Cell-Lung-Cancer (NSCLC) cells mediated by Gli through regulation of cyclin expression. Moreover, increased Shh expression in NSCLC might be related to cancer progression mediated by cancer stroma-associated fibroblast.^299^

Researchers found that in patients with lung adenocarcinomas tumors and lung squamous cell carcinomas had higher Gli expression, accompanied by significantly lower expression of an EMT marker E-Cadherin. Inhibition of HH signaling pathway in vivo decreased tumor growth and induced E-Cadherin expression.^300,301^ Epidermal growth factor receptor tyrosine kinase inhibitors (EGFR-TKIs) are widely used as first line therapy for NSCLC. Upregulation of HH signaling pathway resulted in EGFR-TKIs resistance by EMT induction. Blockade of HH signaling increased sensitivity to EGFR-TKIs in NSCLC cells.^302^ HH signaling pathway drives lung adenocarcinoma (LAC) cells growth under stress conditions such as serum-starvation.^303^ Moreover, microRNA (miRNA) disorders might be related to cell cisplatin resistance in LAC through targeting Gli2.^304^ Besides, researchers found that Shh^+^ NSCLC cells produced full-length Shh protein on the membranes of these cells. Shh^+^ cells exhibited chemo-resistance and showed proliferation and migration stimulatory effect on Shh^-^ cells in a paracrine manner.^305^

### Hematological malignancy

It has been found that HH components are aberrantly activated in a variety of hematological malignancies including multiple biological behavior. HH signaling pathway was upregulated in CD34^+^ hematopoietic cells from patients with chronic myelomonocytic leukemia (CML).^306,307^ HH signaling pathway is increased in BCR-ABL^+^ progenitor cells in CML through enhanced expression of Smo. Smo is responsible for the downregulation of microRNA-326 in patient with CML.^308,309^ Inhibiting Gli2 abolishes dormancy in human leukemia stem cells.^310^

Gli expression can be recognized as a prognostic indicator for acute myeloid leukemia (AML) and elevated Dhh plasma level can be detected in AML patient.^311^ In a murine model of myelodysplastic syndrome (MDS), HH signaling pathway activation led to leukemic transformation to AML by acquiring self-renewal potential.^312^ Stromal cells show potential of supporting activity for proliferation of leukemic cells and the expression of human Hhip in AML/MDS-derived stromal cells was markedly lower than stromal cells from healthy individuals.^313^ HH/Gli-1 also plays a key role in resistance to radiation, and that inhibition by LDE225 precipitates a radiation-resistant cell line overcoming radioresistance.^314^ HH signaling pathway has important impact on B-cell in chronic lymphoblastic leukemia, acute lymphoblastic leukemia (ALL) and multiple myeloma.^315–317^ HH signaling pathway is associated with progression of these diseases.^318^ Targeting HH signaling pathway holds promising therapeutic effects.

### Thyroid cancer

A total of 80% of thyroid cancers occur as papillary thyroid carcinomas (PTCs).^319^ A majority of Thyroid tumor specimens are positive for HH signaling pathway component (Shh, Ptch, Smo, and Gli) while inhibition of HH signaling pathway reduced proliferation of thyroid tumor cell lines.^320^ Shh enhances cell motility and invasiveness of anaplastic thyroid carcinomas.^321^ Moreover, Shh signaling pathway is important to PTCs occurrence and progression.^322,323^ It also regulates CSC self-renewal and radiosensitivity in anaplastic thyroid carcinomas cell lines by Snail expression.^324^ Ma et al. proposed that the loss of primary cilia has been linked to the malignant transformation in thyrocyte.^325^

### Basal cell carcinoma

As the most common skin malignant tumor, BCC is most likely to occur in the skin of head and face.^326^ Multiple BCC is a major symptom of Gorlin syndrome. 90% of sporadic BCC cases carry somatic mutations in Ptch, others have gain of-function mutations in Smo, leading to over-activation of the pathway.^327,328^ Overexpression of Ptch1 and Gli1 were observed in BCC compared with normal epidermal tissue and enhanced Shh immunoexpression was found in the aggressive BCC.^329,330^ Interestingly, Gli1 was specifically upregulated in BCC, while the other skin malignancies such as squamous cell carcinoma showed no Gli1 expression.^331^

Intrafollicular epidermal stem cells are reported to induce BCC formation through HH signaling pathway due to their enhanced self-renewing ability.^332^ The tumor epithelium of human BCC with Smo inhibitor resistance possesses reduced primary cilia. Loss of primary cilia might be related to HH signaling pathway inactivation and upregulated RAS/MAPK pathway in resistant BCCs.^333^ A lower cilia count in the primary lesion might be correlated with BCC recurrence according to a preliminary study.^334^ Whitson et al. reported non-canonical hedgehog pathway activation also drives drug resistance of Smo inhibitor in BCC.^335^

### Triple negative breast cancer

There were 2.3 million new cases of female breast cancer (BC) in 2020, surpassing lung cancer as the most common cancer worldwide.^185^ Among all BC subtypes, 10% are triple negative breast cancer (TNBC). Poor prognosis and the lack of efficient targeted therapy make TNBC treatment most challenging.

Multiple studies have shown that HH signaling pathway plays an essential role in normal mammary gland development.^336^ HH signaling pathway mediates ductal morphogenesis of the mammary gland during puberty and remains downregulated in normal adult mammary tissue.^336,337^

HH signaling pathway in relation to the aggressive biological behavior of TNBC, including cancer migration, invasion and angiogenesis, has also been discussed.^338^ Somatic mutations of HH signaling pathway components have been shown to be relatively rare in BC, where ligand-dependent HH signaling pathway activation played a more fundamental role in the pathogenesis of TNBC.^339^ Moreover, expression of Shh, Dhh, Ptch1, Gli1 has been observed to be upregulated in BC tumor tissue.^340^ Smo and Gli1 in TNBC tissue is also significantly increased compared to those of non-triple-negative BC.^341^ Furthermore, TNBC patients with higher Shh expression have worse overall survival.^342^

HH signaling pathway promotes TNBC progression in an autocrine manner, while promoting tumor vascularization via regulation of vascular endothelial growth factor receptor 2 (VEGFR2) expression in a paracrine manner.^343^ Another possible mechanism of TNBC chemoresistance is HH signaling pathway upregulated ATP binding cassette (ABC) transporters and other target genes.^338^ TNBC cells may also harbor dysfunctional stemness pathways compared to non-triple-negative BC cells.^338^ TNBC associated fibroblasts can activate HH signaling pathway in a paracrine manner and enhance tumor cell growth.^344^ In addition, HH-activated CAFs have been shown to form a niche for chemo-resistant TNBC stem cells and target HH-activated CAFs via Smo inhibitors, which benefits patients suffering from TNBC.^345^ Exposure to chemotherapeutics agents of TNBC cell lines results in the release of Shh and HH signaling pathway activation, leading to an increase in the stemness marker of TNBC stem cells, which may explain the chemoresistance and recurrence of TNBC.^346^ The above evidence may illustrate one possible way that could relate to the aggressiveness and difficulty in treating TNBC by HH signaling pathway.

### Rhabdomyosarcoma

Rhabdomyosarcoma (RMS) is known to be the most common type of soft tissue sarcoma in children whose cells tend to undergo myogenic differentiation.^347^ Two major subtypes of this condition are embryonal subtype of RMS (ERMS) and alveolar subtype of RMS (ARMS).^348^

The relationship between RMS and HH signaling pathway was first identified in mice heterozygous for Ptch1 as these mutants exhibited a high incidence of ERMS.^349^ The consistent activation of the HH pathway has been associated with RMS, though there have been relatively few RMS cases in patients with Gorlin syndrome.^211^ According to the Children’s Oncology Group, 18% of patients with fusion-negative RMS harbor a mutation in HH signaling pathway.^350^

Controversy is still prevalent pertaining to the exact mechanism of the constitutive activation of HH signaling pathway in RMS. Overexpression of HH signaling pathway components, such as Gli1 and Ptch1, has been detected in RMS patient samples and human RMS cell lines.^351^ However, activating mutations of HH signaling pathway reported to date only account for a small subset of patients.^347^ A particular study proposed a different model of RMS in HH signaling pathway activation, where a wide distribution of Dhh and Ihh ligands were detected by immunohistology in RMS samples, in which Shh was only found to be expressed in a small set of RMS cell lines. These findings demonstrated the potential mechanism of the ligand-dependent activation of HH signaling pathway in RMS.^352^ Hatley et al. utilized *aP2-Cre; Smo*^*M2*/+^ mutant mice and discovered that the activated HH signaling pathway in the adipose lineage led to ERMS in mice.^353^ Fu et al. reported that primary cilia play a central role in muscle differentiation. Either ciliogenesis or HH signaling pathway are dysregulated in RMS.^354^

Targeting HH signaling pathway in RMS has demonstrated therapeutic potential in in vitro studies, and its inhibition contributes to the suppression of cell invasion and self-renewal in RMS cell lines.^355,356^ Meanwhile, upregulation of Gli1 contributes to drug resistance.^357^

However, the pathogenesis of RMS as well as the oncogenic role of HH signaling pathway remains unclear, and further studies should be urgently conducted in order to develop effective treatment options for RMS

### Gastric cancer

Gastric cancer (GC) deaths ranks fourth among all cancers and has led to more than 760,000 deaths in 2020.^185^ Although the incidence of GC decreases annually, gastric cancer continues to be fatal, with a sharp shortened survival.^358^

In stomach, HH signaling pathway is essential for differentiation and maturation of gastric epithelial cells under physiological conditions.^359^ Shh is highly expressed in gastric parietal cells for gastric acid and gastrin production, illustrating that HH signaling pathway is indispensable for normal digestive function.^360^

HH signaling pathway links progression from chronic inflammation to cancerous lesions.^361^ *Helicobacter pylori* (*H pylori*) infection is one of the main causes of chronic gastritis and is a significant risk factor for gastric cancer. Multiple studies have reported that chronic *H pylori* infection can interfere in the balance of HH pathway activity within gastric tissue.^360^ *H pylori* may also contribute to gastric atrophy and intestinal metaplasia for increased potential of tumorigenesis.^362^ Mice with parietal cell-specific deletion of Shh were not observed to develop gastritis when infected by *H pylori*, and Shh in gastric mucosa was considered to act as a chemoattractant for macrophages.^363^

Shh has been found to be overexpressed in resected GC samples compared to that of adjacent normal tissue.^364^ Specifically, Shh expressing in *H pylori* positive patients with early GC was noted to be significantly higher than *H pylori* negative controls.^365^ The increased expression of Shh and Gli1 was also shown to be significantly correlated with tumor staging and tumor aggressiveness, suggesting a worse overall survival for patients suffering from GC.^193,366^

Shh promotes gastric cancer cell proliferation and the survival of lines, which suggests an autocrine progression of gastric cancer. It also regulates gastric cancer migration and invasion of gastric cancer cell lines through EMT.

Shh-Gli1 signaling was activated in CD44^+^ gastric cancer stem cells, which was responsible for drug resistance in advanced GC. Moreover, Gli interacts with the promoter of ABCG2 and regulates its expression in gastric cancer stem cells.^367^ Accordingly, targeting HH signaling pathway may improve GC chemosensitivity.^368,369^ Koh et al. investigated PDL-1 expression mediated by Gli in gastric cancer organoids, which may relate to immune evasion in GC.^370^

### A brief summary on HH signaling pathway in cancer

HH signaling pathway participates in multiple functions in a variety of tumors. According to immunohistology, early exploration of HH signaling pathway was concentrated on the expression and distribution of the HH pathway components in tumor tissue. One potential reason for controversies in early studies that examined the expression patterns of HH signaling pathway proteins in cancer could be differential tumor samples analyzed.^371^ In light of the rapid understanding of tumor biology, additional features of HH signaling-associated cancers have been determined. Cancer stem cells and the hedgehog pathway are closely related to cancer relapse and cancer resistance to anticancer therapy. In addition, the mechanism of HH signaling pathway activation in cancer is multifactorial, demonstrating that canonical pathway activation and noncanonical pathway activation may exist in a single form of cancer.^372^ Similarly, ligand-dependent and ligand-independent activation may promote cancer progression cooperatively. Therefore, further studies should focus on the precise mechanism of HH signaling pathway in cancer for better therapeutic targets.

## Targeted therapies of HH signaling in cancers

### Introduction of targeted therapies

**Fig. 5:**
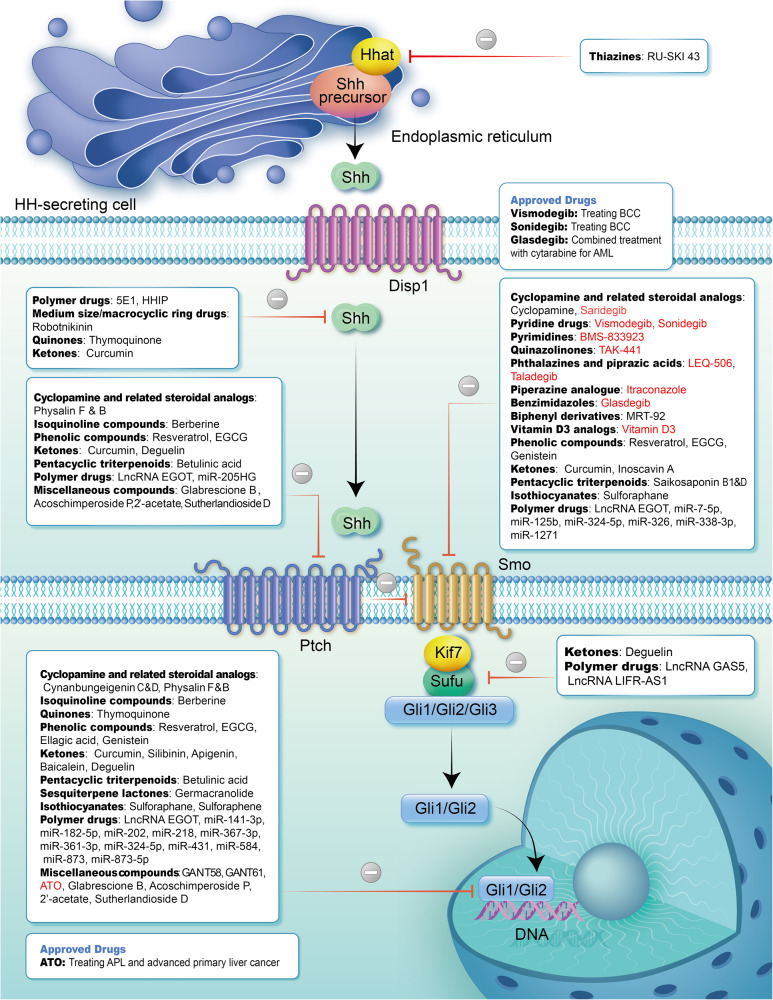
Inhibitors targeting HH signaling pathway. In this figure, we mainly show the inhibitors targeting HH signaling pathway, classified according to the properties of the compounds, and the inhibitors in ongoing or completed clinical trials are shown in red. Approved drugs are individually identified, including their indications. BCC basal cell carcinomas, AML acute myeloid leukemia, APL acute promyelocytic leukemia

Recent breakthroughs have revealed the carcinogenic function of HH signaling pathway, which makes it an interesting target for cancer treatment. Drugs related to HH signaling pathway target Hhat, Shh, Ptch, Smo, Sufu and Gli1 (Fig. 5). These drugs targeting HH signal transduction can be divided into small molecule inhibitors, natural chemicals, lncRNAs and monoclonal antibodies. Among them, small molecule inhibitors are the most widely studied, usually referring to chemical compounds with molecular weight less than 1 kDa. These drugs have significant advantages: (1) small molecular weight brings more obvious permeability to tissues and cells; (2) there are various forms of drug delivery; (3) oral tolerance and bioavailability are good.

In 2000, the first drug targeting HH signaling pathway, arsenic trioxide (ATO), was approved for the treatment of acute promyelocytic leukemia (APL).^373^ ATO not only inhibits HH signaling pathway, but also targets other signaling that involved in the development and progression of APL. The first Food and Drug Administration (FDA) approved drug specifically designed to target HH signaling pathway is vismodegib (GDC-449), which was approved for the treatment of BCC in 2012.^374^ Since then, sonidegib and glasdegib have been approved for clinical treatment of cancer.^375,376^ Although vismodegib and sonidegib have achieved satisfactory outcome in the treatment of locally advanced or metastatic BCC, frequent Smo mutations lead to increased drug resistance.^377^ Therefore, recent studies have revealed that new HH signaling pathway inhibitors and reasonable multi target drugs are applied together to solve this problem, including taladegib, LEQ-506 and TAK-441, which are not sensitive to drug-resistant D473H mutations.^378–380^ In addition, some studies on Hhat, Shh and Gli1 inhibitors are also expected to inhibit Smo resistant cells.

### Hhat inhibitor

Hhat catalyzes the completion of palmitoylation of HH proteins to maintain their stability and normal activity.^381^ Inhibitors targeting Hhat may therefore block HH protein-mediated pathway activation. Current inhibitor targeting Hhat was RU-SKI 43. Petrova et al. carried out a high throughput screen using a peptide-based assay and identified Hhat specific inhibition by RU-SKI 43  both intracellularly and in vitro.^382^ They further investigated the potential effect of RU-SKI 43 in cancer. The results suggested that RU-SKI 43 was able to reduce Gli1 activation, Akt and mTOR pathway activity and tumor growth in vitro in pancreatic cancer cell proliferation and in vivo in xenograft models by inhibiting Hhat.^383^ It should be noted that the inhibition effect of RU-SKI 43 was not mediated through Shh protein.^383^ Another study showed similar observations that RU-SKI 43 treatment reduced the proliferation of estrogen receptor positive breast cancer cells, showing a dose-dependent effect.^384^ Overexpression of Hhat protein was able to rescue this inhibition, but the addition of exogenous recombinant Shh protein did not.^384^ The lack of Hhat inhibitor studies may be due to the need to use radiolabeled fatty acid substrates to measure activity, which has limitations in terms of throughput, cost and safety.^385^ Recently, Andrei et al. proposed an acylation-coupled lipophilic polarization induction assay. They applied this method to discover the potent Hhat inhibitor IMP-1575.^385,386^ Future studies will be needed to reveal the role of IMP-1575 in anticancer therapy.

### Shh inhibitor

Shh proteins bind to and inhibit Ptch1 receptor, which could unblock Ptch1 for Smo proteins and cause typical HH signaling pathway. Inhibitors targeting Shh proteins may therefore block Shh protein-mediated signaling pathway activation. Inhibitors targeting Shh include 5E1, Hedgehog-interacting protein (Hhip) and Robotnikinin.

### 5E1

5E1 is a monoclonal antibody targeting Shh and blocks HH signaling pathway by competing with Ptch1 for the Shh binding site.^387^ Treatment of colorectal cancer cell lines with 5E1 reduced the expression of HH signaling pathway target genes (Gli1, Ptch1, Hip1, Muc5ac) as well as cell cycle protein D1 and mediated the reacquisition of epithelial-like features in cells.^388^ 5E1 was also shown to inhibit the self-renewal capacity and chemoresistance of gastric cancer stem cells, demonstrating a potential role for 5E1 in enhancing chemotherapeutic sensitivity.^389^ In the non-Smo protein-mediated HH signaling pathway, 5E1 may have a more satisfactory effect. Both cyclopamine and 5E1 could inhibit chronic lymphocytic leukemia (CLL) cells in B-CLL patients.^390^ Stromal cells could induce activation of the paracrine pathway and activated ERK directly via Ptch1 receptor in CLL cells without Smo.^390^ In this case, cyclopamine treatment was suboptimal, while 5E1 could completely eliminate ERK phosphorylation.^390^

### Hhip

Hhip is a negative regulator of HH signaling pathway that prevents Ptch1 from binding Shh by binding the cholesterol fraction covalently linked to Shh through the N-terminal.^391^ Abnormal expression of Hhip has been associated with cancer development. For example, hypermethylation of the Hhip gene promoter has been found in several tumor cell lines, including HCC, pancreatic cancer, MB, and various gastrointestinal tumors.^392–395^ Down-regulation of Hhip expression using siRNA resulted in a significant increase in colon cancer cell growth and invasion in vitro.^395^ Therefore, adjustment of Hhip level allows regulation of cancer development. Yu et al. used a lentiviral vector to deliver Hhip. The result showed that gastric cancer cells in the Hhip group had reduced proliferation, migration and invasion compared to the control.^396^ Interestingly, Hhip overexpression significantly reduced its de novo promoter methylation level in gastric cancer cells.^396^

### Robotnikinin

Robotnikinin is a small molecule that targets Shh-N protein. It has the following features: inhibits HH signaling pathway in a concentration dependent manner; does not show inhibitory activity in the absence of Ptch1 receptors; does not compete with cyclopamine.^397^ Robotnikinin reduced Gli2 mRNA levels in human keratin forming cells and in primary human synthetic skin tissue.^397^ There is currently lack of other studies revealing the potential application of robotnikinin in the treatment of cancer.

### Ptch activator

Ptch is an inhibitor of HH signaling pathway. Without Shh ligand, Ptch plays a regulatory role by inhibiting the Smo protein.^398^ Ptch protein silencing or gene deletion is related to the development of many cancers, such as odontogenic keratocyst, BCC and plexiform fibromyxoma.^213,399,400^ Therefore, activators targeting Ptch can prevent cancer development by inhibiting Smo protein. At present, some natural compounds have been found to increase Ptch1 expression, for example, the combination of epigallocatechin gallery (EGCG) and theaflavin (TF) can reduce the expression of Gli1 and Smo while increase the expression of Ptch1 in mouse liver cancer cells.^401^

### Smo inhibitor

Within HH signaling pathway, Smo protein is often utilized as the drug target because of (1) the ability to transduce downstream signaling pathways in cancers with a loss of function of Ptch1, (2) cancer-causing capacity in the presence of aberrant expression, and (3) the hydrophobic structural transmembrane helical domain capable of binding a variety of small-molecule drugs. The inhibitors targeting Smo mainly include cyclopamine, and cyclopamine derivatives such as vismodegib, sonidegib, etc.

### Cyclopamine

Cyclopamine is a steroidal alkaloid metabolite produced by *Veratrum californicum*, which has been shown to bind and inhibit the activity of Smo proteins.^402,403^ Cyclopamine may inhibit the development of several cancers by affecting HH signaling pathway.  Cyclopamine is able to block HH signaling pathway in vitro, which in turn affects the aggressiveness and motility of human HCC cells.^404,405^ On the other hand, in an in vivo study, Jeng et al. observed a statistically significant reduction in tumor size in mice with HCC (0.152 ± 0.219 cm^2^ vs. 0.003 ± 0.009 cm^2^) following a 2-week cyclopamine injection at a dose of 30 mg/kg/day.^406^ Cyclopamine has also been shown to inhibit the self-renewal and invasive capacity of cancer stem cells in pancreatic cancer and glioblastoma.^407,408^ Unfortunately, the poor oral bioavailability and specificity of cyclopamine limit its applications.^409^ Under this circumstance, semi-synthetic cyclopamine derivatives such as vismodegib, sonidegib and saridegib have been developed, which show better oral bioavailability and perform well in several clinical trials. Of note, cyclopamine may still function as an anti-cancer agent for sensitization to radiotherapy. For instance, Tsai et al. showed that cyclopamine in combination with radiation therapy resulted in a 67% reduction in the average size of in situ tumors compared to radiation therapy alone.^410^

### Vismodegib (GDC-0449)

Vismodegib, a second generation cyclopamine derivative, was approved by the FDA in 2012 as the first Shh inhibitor for BCC treatment.^374,411^ Vismodegib achieved satisfactory results in clinical trials in patients with locally advanced and metastatic BCC. After 21 months of daily oral administration of 150 mg vismodegib, the objective remission rate was 47.6% for locally advanced BCC and 33.3% for metastatic BCC, with a median remission and progression-free survival of 9.5 months months.^412,413^ Several phase I and phase II studies have investigated the use of vismodegib in detail, for instance, vismodegib in the treatment of TNBC, multiple BCC, recurrent/refractory MB, and keratinized cystic odontogenic tumors with significant therapeutic benefit.^345,414–416^ In 2016, Jacobsen et al. evaluated the effect of vismodegib for BCC by meta analysis.^417^ Data from eight studies with a total of 744 patients showed that: objective response to vismodegib for locally advanced BCC had a weighted average of 64.7% and complete response averaged 31.1%. The objective response for metastatic BCC was 33.6% and complete response averaged 3.9%.^417^ This meta-analysis showed that vismodegib was identified to have a significant and consistent effect on the median duration of therapy (35.8 weeks) of locally advanced BCC and metastatic BCC. Vismodegib, on the other hand, was not effective in the treatment of metastatic pancreatic cancer, lymphoma, chronic leukemia, and advanced osteosarcoma.^418–420^ Therefore, more comprehensive clinical trials are needed to validate vismodegib in different populations. Several clinical trials of vismodegib are currently underway as monotherapy as well as in combination therapy for leukemia, meningioma and chondrosarcoma (Table 3). Adverse events associated with vismodegib include muscle cramps, taste disturbances, weight loss, hair loss, and weakness.^421^ These conditions often subside spontaneously within 4 weeks, but the management of adverse reactions should not be neglected.^421^

**Table 3:** Summary of drugs in clinical trials targeting canonical HH signaling in cancer

Drugs	Target protein	NCT	N	Status	Phase	Cancer
Vismodegib						
Vismodegib	Smo	NCT02436408	35	Completed	Phase IV	BCC
		NCT01201915	74	Completed	Phase II	BCC
		NCT01267955	45	Active	Phase II	Chondrosarcoma
		NCT01367665	1232	Completed	Phase II	BCC
		NCT01700049	28	Completed	Phase II	BCC
		NCT01815840	229	Completed	Phase II	BCC
		NCT00739661	104	Completed	Phase II	OC
		NCT02366312	2	Completed	Phase II	KCOT
		NCT03052478	10	Completed	Phase II	Stomach neoplasms
Vismodegib/Chemotherapy/Other	Smo	NCT01878617	660	Active	Phase II	MB
Vismodegib/GSK2256098/Capivasertib/Abemaciclib	Smo/ FAK/ AKT / CDK	NCT02523014	124	Recruiting	Phase II	Progressive meningiomas
Vismodegib+ Chemotherapy	Smo	NCT01835626	24	Completed	Phase II	Locally advanced BCC; Skin cancer; CM
Vismodegib+ Chemotherapy	Smo	NCT02694224	40	Recruiting	Phase II	TNBC
Vismodegib+ Pembrolizumab	Smo + PD-1	NCT02690948	16	Completed	Phase I-II	Metastatic or unresectable BCC
Vismodegib+ Ribavirin+ Decitabine	Smo	NCT02073838	23	Completed	Phase II	AML
Vismodegib/Other targeted drugs	Smo	NCT02091141	670	Completed	Phase II	Neoplasms
Vismodegib/Other targeted drugs	Smo	NCT03158389	350	Recruiting	Phase I-II	Glioblastoma
Vismodegib/Other targeted drugs	Smo	NCT03297606	720	Recruiting	Phase II	NHL; MM; Advanced solid tumors
Vismodegib+ Bevacizumab	Smo + VEGF	NCT00636610	199	Completed	Phase II	Metastatic CRC
Vismodegib+ Gemcitabine Hydrochloride	Smo	NCT01064622	118	Completed	Phase I-II	PC
Vismodegib+ Gemcitabine Hydrochloride+ nab-Paclitaxel	Smo	NCT01088815	98	Completed	Phase II	PC
Vismodegib+ RO4929097	Smo + Gamma-Secretase	NCT01154452	78	Completed	Phase I-II	Advanced or metastatic sarcoma
Vismodegib+ Gemcitabine Hydrochloride	Smo	NCT01195415	25	Completed	Phase II	PC
Sonidegib						
Sonidegib	Smo	NCT03534947	10	Recruiting	Phase II	BCC
		NCT01708174	22	Completed	Phase II	MB
		NCT00961896	18	Completed	Phase II	BCNS
		NCT01125800	76	Completed	Phase I-II	MB
		NCT01350115	10	Completed	Phase II	BCNS
Sonidegib+ Lenalidomide	Smo + Cereblon	NCT02086552	28	Completed	Phase II	Recurrent/refractory PCM
Sonidegib+ Ribociclib	Smo + CDK4/6	NCT03434262	68	Active	Phase I	Refractory or recurrent MB
Sonidegib+ Radiotherapy+Chemotherapy	Smo	NCT04402073	205	Recruiting	Phase II	MB
Saridegib						
Saridegib	Smo	NCT02762084	17	Completed	Phase II	BCNS
		NCT02828111	36	Completed	Phase II	BCC
Saridegib+ Gemcitabine	Smo	NCT01130142	122	Completed	Phase I-II	PC
Glasdegib						
Glasdegib	Smo	NCT01841333	31	Completed	Phase II	AML
		NCT01286467	23	Completed	Phase I	Solid tumors
		NCT01842646	35	Completed	Phase II	MDS; CML
Glasdegib+ Temozolomide Oral Capsule	Smo	NCT03466450	75	Active	Phase I-II	Glioblastoma
Glasdegib+ Chemotherapy	Smo	NCT01546038	255	Completed	Phase II	AML
Glasdegib+ Other targeted drugs	Smo	NCT03390296	50	Completed	Phase I-II	AML
Glasdegib+ Azacitidine	Smo	NCT04842604	15	Completed	Phase III	AML; MDS; CML
Taladegib						
Taladegib+ Chemotherapy	Smo	NCT02784795	94	Completed	Phase I	Solid tumor; BC; CC; CCC; STS
TAK-441						
TAK-441	Smo	NCT01204073	34	Completed	Phase I	Nonhematologic malignancies
Itraconazole						
Itraconazole	Smo	NCT01108094	29	Completed	Phase II	BCC
		NCT01787331	21	Completed	Phase II	Prostate adenocarcinoma
		NCT02354261	38	Completed	Phase II	BCC
Itraconazole+ AZD9291	Smo + EGFR	NCT02157883	39	Completed	Phase I	Advanced/inoperable NSCLC
Itraconazole+ volasertib	Smo + PLK1	NCT01772563	28	Completed	Phase I	Neoplasms
Itraconazole+ Other drugs	Smo	NCT02770378	10	Completed	Phase I	Glioblastoma
Vitamin D3						
Vitamin D3	Smo	NCT02553447	197	Active	Phase I	CLL; NHL
Vitamin D3+ Photodynamic Therapy	Smo	NCT03483441	37	Active	Phase I	BCC; BCNS
Vitamin D+ Rituximab	Smo + CD20	NCT03078855	211	Active	Phase III	NHL
ATO						
ATO + GO	Gli + CD33	NCT00274781	30	Completed	Phase II	Advanced MDS
ATO + GO + ATRA	Gli + CD33	NCT01409161	151	Recruiting	Phase II	APML

*BCC* Basal cell carcinoma, *OC* ovarian cancer, *BCNS* Basal cell nevus syndrome, *MB* medulloblastoma, *KCOT* keratocystic odontogenic tumor, *CRC* colorectal cancer, *CM* cutaneous malignancy, *TNBC* triple negative breast cancer, *AML* acute myeloid leukemia, *PCM* plasma cell myeloma, *NHL* non-Hodgkin lymphoma, *PC* pancreatic cancer, *MM* multiple myeloma, *CML* chronic myelomonocytic leukemia, *MDS* myelodysplastic syndrome, *EC* esophageal cancer, *BC* breast cancer, *CCC* cholangiocarcinoma, *CC* colon cancer, *STS* soft tissue sarcoma, *NSCLC* non-small cell lung cancer, *CLL* chronic lymphocytic leukemia, *APML* acute promyelocytic leukemia

### Sonidegib (LDE225)

Sonidegib (LDE225) is a Smo antagonist found by high-throughput screening in vitro.^422^ In vivo and in vitro studies found that sonidegib can effectively reduce epithelial mesenchymal transformation and invasion potential of various types of cancer, such as glioblastoma, PCa and renal cell carcinoma.^251,423,424^ A phase II clinical trial proved that sonidegib was administered with 200 mg and 800 mg respectively, the objective remission rates of locally advanced BCC were 57.6% and 43.8%, metastatic BCC were 7.7% and 17.4%, and the incidence of adverse events in the 200 mg group was lower.^425^ Another phase II clinical trial yielded the similar results.^426^ Based on this, FDA approved sonidegib for the treatment of locally advanced BCC in 2015.^375^ A meta-analysis of Shh inhibitors for BCC included 22 studies from 2009–2022 and showed overall response rates (ORRs) of 50.1% for sonidegib compared with 68.5% for vismodegib.^427^ This indicates that the majority of patients receiving sonidegib and vismodegib achieved promising treatment outcomes. In recent years, some clinical studies have focused on applying sonidegib to other cancers, such as MB, ovarian cancer, breast cancer, etc.^428–430^ Sonidegib showed ideal therapeutic effect and safety in the treatment of these cancers. Sonidegib related adverse events included muscle spasms, taste disorders, nausea, alopecia, and elevated creatine kinase levels.^431^ These reactions are often mild, but long-term adverse reactions may also lead to the decline of patients’ quality of life and drug withdrawal.^431^ Compared with vismodegib, patients receiving sonidegib treatment had lower overall adverse events and occurred more slowly.^432^ At present, several clinical trials of sonidegib as a single therapy and a combination therapy are under way, mainly for the treatment of MB (Table 3).

### Saridegib (patidegib, IPI-926)

Saridegib (patidigib, IPI-926) is a semi synthetic derivative of cycloparamide in Smo inhibitor, which has significantly improved drug characteristics, efficacy and good pharmacokinetic characteristics.^433^ In vivo and in vitro studies show that saridegib can effectively inhibit the proliferation and invasion of chondrosarcoma, serous ovarian cancer, osteosarcoma, acute lymphocytic leukemia and other cancer.^434–437^ It is worth noting that saridegib also shows a prominent inhibitory effect on the drug-resistant cells obtained by D473H point mutation after the treatment of MB with vismodegib.^438^ A phase I study used saridegib and FOLFIRINO (5-fluorouracil, leucovorin, irinotecan, oxaliplatin) to treat 15 patients with advanced pancreatic cancer. Four out of five patients treated with saridegib monotherapy observed a continuous decrease in CA19-9 (26.9–97.7%).^439^ In addition, saridegib and cetuximab also showed anti-tumor activity in the phase I study on the treatment of recurrent/metastatic head and neck squamous cell carcinoma.^440^ The common adverse reactions of saridegib treatment are fatigue, nausea, muscle spasm, liver dysfunction and alopecia.^441^ These adverse reactions are similar to other HH signaling pathway inhibitors and may be caused by inhibition of the same pathway.

### Glasdegib (PF-04449913)

Glasdegib is a selective small molecule inhibitor that binds to Smo protein.^442^ A randomized phase II clinical trial showed that compared with cytarabine alone, glasdegib combined with cytarabine can effectively treat AML patients who are not suitable for intensive chemotherapy or have high-risk MDS (median survival, 8.8 vs 4.9 months; 12 month survival, 59.8% vs 38.2%), with better safety and tolerance.^443^ Based on this study, FDA approved the combined treatment of glasdegib and low-dose cytarabine for newly diagnosed AML patients who are not suitable for intensive induction chemotherapy in 2018.^376^ In addition, the phase I study of glasdegib in patients with advanced solid tumors shows that it can keep the patient’s condition stable for a long time.^444^ At present, glasdegib is conducting further clinical trials, including evaluating whether it can effectively treat AML, MDS or CML patients who have not been treated before with or without cytosine arabinoside (NCT04842604) and whether the combination of glasdegib and temozolomide can effectively treat glioblastoma (NCT03466450). Further clinical research will help to reveal the safety and effectiveness of glasdegib in cancer treatment.

### Taladegib (LY2940680)

Taladegib binds to the extracellular end of the Smo transmembrane spiral bundle to inhibit the transmission of HH signal transduction.^58^ The effect of taladegib is not affected by D473 mutation, and it can effectively treat tumor cells resistant to vismodegib.^378^ Phase I clinical trial proved that taladegib has acceptable safety in the treatment of locally advanced and metastatic BCC patients. The common adverse events are taste disorder, fatigue, nausea and muscle spasm.^445^ It is worth noting that another phase I study showed that Notch inhibitor crenigacestat combined with taladegib was poorly tolerated in the treatment of patients with advanced or metastatic solid tumors, showing disappointing clinical efficacy.^446^ The reason why the combination therapy is not ideal may be due to the heterogeneity of the patient population or the history of systemic therapy.^446^ Further research on taladegib single drug therapy or combination therapy will fully reveal its safety and therapeutic effect.

### XL-139 (BMS-833923)

XL-139 (BMS-833923) is a Smo inhibitor, which has been proved to inhibit tumor in many in vitro and in vivo studies. For example, XL-139 can reduce the activity of HH signaling pathway, reduce cell proliferation, while induce apoptosis of esophageal cancer cells in vitro.^447^ XL-139 can inhibit amyloblastoma cells with SMO-L412F mutation resistant to vismodegib.^448^ XL-139 combined with gemcitabine can significantly inhibit the growth of CCC in the subcutaneous xenotransplantation model of mice.^276^ Two clinical studies were designed to explore the effect of XL-139 combined with dasatinib in the treatment of leukemia, but the trial failed to proceed as expected (NCT01218477, NCT01357655). Therefore, further research is needed to reveal the safety and effectiveness of XL-139.

### LEQ-506

LEQ-506 is a small molecule inhibitor of Smo, and there are few relevant studies at present. For D473H mutant cells resistant to vismodegib and sonidegib, LEQ-506 was not affected and showed inhibitory effect.^379^ In addition, in vivo study has proved that LEQ-506 at 1% concentration can effectively inhibit the expression of Gli1 (80–90%) in the skin cells of depilated mice.^449^

### TAK-441

TAK-441 is an oral small molecule Smo inhibitor. Preclinical studies showed that its 50% inhibitory concentration (IC50) on Gli1 transcriptional activity was 4.4 nmol/L.^450^ Similar to LEQ-506, TAK-441 can be used to treat D473H mutants resistant to vismodegib.^380^ In vivo studies have proved that TAK-441 can inhibit Gli1 mRNA expression and tumor progression in castration resistant PCa and xenotransplantation of pancreatic tumor in mice.^451,452^ A phase I clinical trial used TAK-441 to treat 34 patients with solid tumors, including colorectal cancer (26%), BCC (21%) and pancreatic cancer (9%). The results showed that TAK-441 was well tolerated and had preliminary anti-tumor activity.^453^ The common adverse reactions of TAK-441 during treatment are taste disorder, fatigue, nausea and muscle spasm, mostly mild to moderate.^453^

### Itraconazole

Itraconazole is a drug to treat systemic fungal infection, especially in patients with low immune function and cancer.^454^ In 2010, it was found that itraconazole could inhibit HH signaling pathway by preventing the accumulation of Smo in primary cilia.^455^ The unique mechanism of action makes itraconazole a feasible choice for the treatment of other HH signaling pathway inhibitors resistance (such as vismodegib). Many in vivo and in vitro studies have proved that itraconazole can effectively suppress the proliferation of cancer cells, including MB, oral squamous cell carcinoma, gastric cancer, malignant pleural mesothelioma, etc.^455–458^ Phase II clinical trial showed that after treatment with itraconazole in 19 patients with basal cells, cell proliferation decreased by 45% (*P* = 0.04), HH signaling pathway activity decreased by 65% (*P* = 0.03), and tumor area decreased by 24%.^459^

### MRT-92

MRT-92 is a small molecule inhibitor based on acyl guanidine or acyl thiourea scaffold, which can bind to the whole 7-transmembrane domain of Smo, and is insensitive to human D473H.^460,461^ When inhibiting the proliferation of rat cerebellar granule cells by more than 50%, the required dose of MRT-92 was 0.3 μM and 3 μM for vismodegib.^461^ Further studies showed that MRT-92 could inhibit the growth of melanoma cells in vitro and xenograft melanoma in mice by inducing DNA damage and G2/M cell cycle arrest.^462^ Another study identified a new BRD4-SOX2 transcription complex, which is related to the non-standard activation of Gli1 in melanoma.^463^ The researchers combined the powerful BRD4 degrading agent MZ1 with MRT-92, showing a synergistic anti-proliferation effect on melanoma cells.^463^ These studies have demonstrated the potential of MRT-92 single drug therapy and multi-drug therapy for melanoma. More studies will help further reveal the safety and efficacy of MRT-92.

### PF-5274857

PF-5274857 is an oral Smo antagonist. In vivo studies have shown that it can effectively penetrate the blood-brain barrier and inhibit the activity of Smo protein in the brain of mice with primary MB, thereby improving the survival rate of animals.^464^ PF-5274857 has potential in the treatment of brain tumors driven by the activated HH signaling pathway or brain metastasis of primary tumors, but there is still a lack of further research.

### Gli inhibitor

The nuclear transcription factor Gli is a downstream protein of HH signaling pathway, and inhibitors that target the Gli protein may block its binding to downstream genes and exert an inhibitory effect on HH signaling pathway.^465^ Although Smo inhibitors have made good progress in the treatment of cancer, the drug-resistant mutations that they cause cannot be ignored.^466^ Studies have shown that inhibitors targeting Gli proteins are effective against primary and secondary drug resistance induced by Smo inhibitors.^467^ Currently, inhibitors targeting Gli include GANT compounds, ATO-like compounds, and Hedgehog pathway inhibitors (HPIs).

### GANT58 and GANT61

The first inhibitors GANT58 and GANT61, which can inhibit Gli protein production, were identified by representative screening in HEK293 cells transiently expressing Gli1 and Gli-dependent luciferase reporter.^468^ GANT58 and GANT61 have no effect on other cancer signaling pathways (e.g. TNF signaling/-NF-κB, glucocorticoid and MAPK pathways), and therefore they are highly selective for Gli.^468^ GANTs can inhibit the proliferation of a variety of cancer cells. For example, GANT58 inhibited spheroid formation and invasion of cervical cancer stem cells undergoing EMT.^469^ Moreover, it showed more potent cytotoxic effects in the face of cyclopamine-resistant T-ALL cells.^470^

Compared to GANT58, GANT61 has a significant inhibitory effect on more types of cancer cells. For example, in vitro studies have shown that GANT61 can inhibit the proliferation and invasiveness of breast cancer, head and neck squamous cell carcinoma, HCC, ovarian plasmacytoma, and PCa.^471–475^ Similar findings were found in in vivo studies, for example, Chang et al. found that the mean volume of cervical cancer in mice after 28 days of GANT61 treatment was significantly reduced compared to the control group (1467.39 ± 403.4 mm^3^ Vs 460.73 ± 91.01 mm^3^).^476^ In addition, GANT61 also inhibited the growth of PCa, MB and rhabdomyosarcoma in vivo.^477,478^ Currently, an increasing number of studies are using GANT61 as a positive control for identifying Gli-specific drugs. Notably, GANT61 under physiological conditions is unstable and rapidly hydrolyzes to aldehydes (GANT61-A) and diamine derivatives (GANT61-D).^479^ GANT61-A lacked biological activity on HH signaling while GANT61-D could inhibit Gli transcription.^479^ This nature limited the applicability of GANT61 and no clinical trials have been conducted so far.

### ATO and darinaparsin (ZIO-101)

ATO is an arsenic compound that has been approved by the FDA as a first-line drug for the treatment of APL, with fewer metastases and longer survival after treatment.^480^ ATO exerts its therapeutic effects by inducing promyelocytic leukemia-retinoic acid receptor multimerization, sumoylation and proteasomal degradation.^481^ In 2010, ATO was validated as a Gli inhibitor, which inhibited Gli transcription and reduced target gene expression by acting directly on the zinc finger structural domain, thereby preventing Gli accumulation in primary cilia and affecting HH signaling pathway.^482,483^ ATO, on the other hand, does not affect the interaction between Gli and DNA.^484^ Yang et al. showed that after 28 months of single ATO treatment, 39 APL patients (86.67%) achieved complete hematological remission, and these patients showed significant downregulation of Gli2 and Smo gene expression.^485^ In addition, several in vitro studies have shown that ATO can inhibit pancreatic cancer, osteosarcoma, rhabdomyosarcoma, malignant pleural mesothelioma and MB cells by acting on Gli.^458,484,486–488^ ATO has been used as a first-line agent in the treatment of APL, but its clinical use in solid tumors is limited by a number of factors, including severe side effects, low drug solubility, and rapid renal clearance.^489^ Pharmacology, drug combinations, and nano-drug delivery systems have all been effective in increasing the therapeutic potential of ATO.^490^

The toxicity of ATO limits its clinical use in solid tumors, so researchers have developed new arsenic analogs to address this issue. Darinaparsin (S-dimethylarsino-glutathione, ZIO-101) is an organic small molecule arsenic compound, and like other arsenic compounds, the arsenic in darinaparsin can bind glutathione and act on the zinc finger structure of Gli protein.^491^ Darinaparsin has better antitumor activity and less systemic toxicity than ATO, and intracellular arsenic levels increase faster, higher, and more consistently than ATO.^492–494^ Furthermore, compared to ATO, darinaparsin showed significantly higher in vitro cytotoxicity and radiosensitizing activity against solid tumor cells under normoxia and hypoxia without affecting normal bone marrow.^495^ Studies have shown that darinaparsin inhibits the proliferation of cancer cells and the growth of prostate tumors in mice by inhibiting Gli2 transcription.^496^ Phase I clinical trials of darinaparsin for refractory solid tumors and phase II clinical trials of Hodgkin’s lymphoma have shown satisfied performance.^497,498^ However, in vivo safety of darinaparsin still requires further study.

### HPIs

HPI is a class of small molecules that directly antagonize Gli, independent of PI3K-AKT, PKA, MAPK and other related pathways.^499^ Each HPI has a unique mechanism of action: (1) HPI-1 inhibits the activation of HH signaling pathway induced by Sufu deletion or Gli overexpression, suppresses Gli protein modification, and affects its function as a transcription factor/cofactor; (2) HPI-2 is less effective against exogenous Gli1 but may interfere with the conversion of full-length Gli2 into a transcriptional activator; (3) HPI-3 inhibits the formation of activated Gli2; (4) HPI-4  acts by disrupting ciliogenesis without affecting Gli2 formation.^499^ In vitro studies have shown that HPI-1 can effectively inhibit the proliferation of small cell lung cancer, breast cancer and other tumor cells.^471,500,501^ In addition, Wei et al. showed that HPI-4 treatment of chondrosarcoma cells significantly decreased the expression of ciliary microtubule transport protein Ift88, Gli protein, and Ptch1 protein, and impaired cell proliferation and invasion ability.^502^ However, the low bioavailability of HPI in vivo due to its high lipophilicity and poor water solubility has limited further studies.^503^

### Other drugs may target HH signaling pathway

In addition to small molecule inhibitors, there are several inhibitors that can target HH signaling pathway, such as natural compounds and LncRNA. Natural compounds are easy to apply, highly available and acceptable therapeutic methods, usually targeting multiple signaling pathways.^504^ It has been found that many natural compounds can target HH signaling pathway and inhibit the proliferation of cancer cells.^504^ Among them, vitamin D3, also known as cholecalciferol, is the most studied and it is formed by 7-dehydrocholesterol after dehydrogenation of cholesterol by ultraviolet radiation. The A-ring of vitamin D3 can directly bind to Smo and inhibit HH signal transduction.^505^ Studies have shown that vitamin D3 can reduce the expression of Gli1 and Ptch and cell proliferation in mouse BCC cell line, and this inhibition is carried out in an independent manner of vitamin D receptor.^506^ At present, several clinical phase I and phase III trials of vitamin D3 are under way to study the role of vitamin D3 monotherapy or combination therapy in non-Hodgkin’s lymphoma and BCC (NCT03078855, NCT02553447, NCT0343441). Since the function of these natural drugs targeting HH signaling pathway has not been fully revealed, we did not include the discussion of these drugs in the current review. Detailed information on natural medicines can be obtained from Table 4.

**Table 4:** Natural compounds and ncRNAs targeting HH signaling in cancers

Name	Features	HH signaling target	Cancer	Model	Effect
Novel natural compounds					
Berberine^517^	GI disease treatment	Gli1, Ptch1	Primary intracranial MB	In vitro and vivo	Tumor growth reduction (37.5%)
Cynanbungeigenin C/Cynanbungeigenin D^518^	Health care drugs	Gli	MB	In vitro and vivo	Inhibition of cell viability and proliferation
Thymoquinone^519^	Anti-oxidant, anti-tumor	Shh, Gli1	PCa	In vitro and vivo	Inhibition of cell viability and proliferation
Resveratrol^520,521^	Anti-aging, anti-tumor	Gli1	GC	In vitro	Cancer cell invasion and metastasis inhibition
		Ptch, Smo	PC	In vitro	Inhibition of cell viability and increase in apoptosis rate
Curcumin^522–525^	Food additives	Gli1	Malignant glioma	In vitro and vivo	Tumor growth reduction (71.4%)
		Shh, Smo, Gli1, Gli2	Lung cancer	In vitro	Inhibition of cell proliferation and increase in apoptosis rate
		Shh, Smo, Gli	PC	In vitro	Inhibition of cell EMT, proliferation and migration
		Shh, Gli1, Ptch1	MB	In vitro	Inhibition of cell viability and increase in apoptosis
EGCG^401,526–528^	Anti-oxidant, anti-tumor	Gli1, Smo	Tongue and liver cancer	In vivo	Limiting histological carcinogenesis
		Ptch1, Smo, Gli1	Liver cancer	In vivo	Limiting histological carcinogenesis
		Smo, Ptch1, Ptch2, Gli	PC	In vitro	Inhibition of cell proliferation and increase in apoptosis
		Gli1, Ptch	Chondrosarcoma	In vitro	Inhibition of cell proliferation and increase in apoptosis
Ellagic acid^529^	Anti-tumor	Gli1, Gli2	PC	In vivo	Tumor growth and metastasis reduction (41.2%)
Saikosaponin B1/Saikosaponin D^530^	Anti-tumor	Smo	MB	In vitro and vivo	Inhibition of cell proliferation and tumor growth
Genistein^531,532^	Estrogen-like activity	Smo, Gli1	BC	In vivo	Tumor growth reduction (68%)
		Gli1	PCa	In vivo	Tumor growth reduction (58.3%)
Glabrescione B^533^		Gli1, Ptch1	MB	In vivo	Tumor growth reduction (63.6%)
		Gli1, Ptch1	BCC	In vitro	Tumor growth reduction (71.4%)
Silibinin^534^	Hepatoprotective drugs	Gli1, Gli2	RCC	In vivo	Tumor growth reduction (64.9%)
Inoscavin A^535^		Smo	CC	In vitro and vivo	Inhibition of cell proliferation and tumor growth
Acoschimperoside P, 2’-acetate^536^		Gli1, Ptch1	PC	In vitro	Inhibition of cancer cell proliferation and migration
Apigenin^537^	Anti-cancer, anti-viral	Gli1	PCa	In vitro	Inhibition of cancer cell proliferation and migration
Baicalein^537^	Treatment of paralysis after cerebrovascular disease	Gli1	PCa	In vitro	Inhibition of cancer cell proliferation and migration
Betulinic acid^538^	Anti-cancer	Gli1, Ptch1	PC and PCa	In vitro	Inhibition of cancer cell proliferation
Deguelin^539^	Insect poisoning, anti-cancer	Gli1, Ptch1, Sufu	PC	In vitro	Inhibition of cell migration and increased apoptosis
Germacranolide^540^		Gli1	PC	In vitro	Inhibition of cancer cell proliferation
Physalin F & B^541^	Anti-inflammatory, immunomodulatory	Gli1, Gli2, Ptch1	PC	In vitro	Inhibition of cancer cell proliferation
Sutherlandioside D^542^	Anti-cancer	Gli1, Ptch1	PCa	In vitro	Inhibition of cancer cell proliferation
Sulforaphene^543^	Anti-cancer	Gli1	BC	In vitro	Cell invasion and metastasis inhibition
Sulforaphane^544^	Anti-oxidant, anti-tumor	Smo, Gli1, Gli2	PC	In vivo	Tumor weight reduction (45.0%)
Vitamin D3^252,506^	Promote calcium and phosphorus absorption	Gli2	RCC	In vivo	Tumor growth reduction (81.4%)
		Gli1	BCC	In vitro and vivo	Tumor growth reduction
NcRNA					
LncRNA GAS5^545^		Sufu	TNBC	In vivo	Increased apoptosis in cancer cells
LncRNA EGOT^546^		Gli1, Smo, Ptch1, Hhip	BC	In vitro and vivo	Inhibition of cell viability and migration capacity
LncRNA LIFR-AS1^547^		Sufu	BC	In vitro and vivo	Inhibition of cancer cell proliferation and migration
miR-7-5p^548,549^		Smo	GC	In vitro	Inhibition of cancer cell proliferation and migration
		Gli3	Bladder cancer	In vitro and vivo	Inhibition of cancer cell proliferation and migration
miR-125b^550^		Smo	MB	In vivo	Inhibition of cancer cell proliferation and migration
miR-141-3p^551^		Gli2	Osteosarcoma	In vitro and vivo	Inhibition of cancer cell proliferation and increased apoptosis in cancer cells
miR-182-5p^304^		Gli1, Gli2	Lung cancer	In vitro	Cell proliferation inhibition and increased sensitivity to cisplatin
miR-202^552^		Gli2	Osteosarcoma	In vitro and vivo	Inhibition of cancer cell proliferation and increased apoptosis in cancer cells
miR-218^553,554^		Gli3	Cervical cancer	In vitro and vivo	Inhibition of cancer cell proliferation and migration
		Gli1	PCa	In vitro	Inhibition of cancer cell proliferation, metastasis and EMT
miR-205HG^555^		Ptch1	EC	In vitro and vivo	Tumor volume reduction (39.4%)
miR-367-3p^556^		Gli1, Gli2	PCa	In vitro and vivo	Inhibition of cancer cell proliferation and migration
miR-361-3p^557^		Gli1, Gli3	Retinoblastoma	In vivo	Inhibition of cancer cell proliferation and migration
miR-324-3p^558^		Gli3	NC	In vitro and vivo	Inhibition of cancer cell proliferation
miR-324-5p^550,559,560^		Smo, Gli1	MB	In vivo	Inhibition of cancer cell proliferation and migration
		Gli1	Glioma	In vitro	Inhibition of cancer cell survival
		Smo, Gli1	MM	In vitro and vivo	Inhibition of cancer cell proliferation and increased apoptosis in cancer cells
miR-326^308,550,561,562^		Smo	MB	In vivo	Inhibition of cancer cell proliferation and migration
		Smo	Glioblastoma	In vitro and vivo	Inhibition of cancer stem cell self-renewal and stemness
		Smo	Osteosarcoma	In vitro and vivo	Inhibition of cancer cell proliferation and migration
		Smo	CML	In vitro and vivo	Inhibition of cancer cell proliferation and increased apoptosis in cancer cells
miR-338-3p^563,564^		Smo	CRC	In vitro	Inhibition of cancer cell proliferation and migration
		Smo	Glioma	In vitro	Inhibition of cancer cell proliferation and migration
miR-431^565^		Gli	Papillary thyroid carcinoma	In vitro and vivo	Inhibition of cancer cell proliferation and migration
miR-506^566^		Gli3	Cervical cancer	In vitro and vivo	Inhibition of cancer cell proliferation and increased apoptosis in cancer cells
miR-584^567^		Gli1	Cervical cancer	In vitro and vivo	Inhibition of cancer cell proliferation and migration
miR-873^568^		Gli1	Cervical cancer	In vitro and vivo	Inhibition of cancer cell proliferation, metastasis and EMT
miR-873-5p^569^		Gli1	GC	In vitro	Inhibition of cancer cell proliferation and migration
miR-1271^570^		Smo	MM	In vitro and vivo	Inhibition of cancer cell proliferation and migration

*BCC* Basal cell carcinoma, *PCa* prostate cancer, *MB* medulloblastoma, *CRC* colorectal cancer, *TNBC* triple negative breast cancer, *PC* pancreatic cancer, *MM* multiple myeloma, *CML* chronic myelomonocytic leukemia, *EC* esophageal cancer, *BC* breast cancer, *CC* colon cancer, *GC* gastric cancer, *RCC* renal cell carcinoma, *NC* nasopharyngeal carcinoma

Non-coding RNA (ncRNA) refers to RNA that does not encode proteins and operates at the RNA level. Recent studies have shown that ncRNA can regulate HH signaling pathway by changing Gli1/2, Smo and Ptch1, such as lncRNA, miRNA and circular RNA (circRNA).^507^ LncRNA can regulate HH signaling pathway through activation or inhibition, which is related to tumor initiation or progression, chemotherapy resistance, recurrence and other processes.^508^ The diagnosis or treatment methods based on lncRNA are promising. In Table 4, we summarized ncRNAs that inhibit cancer development through HH signaling pathway. The existing challenge is how to deliver the specificity of oligonucleotide inhibitors to tumor targets. At present, nano based drug delivery systems are being developed to deliver oligonucleotide inhibitors, such as liposomes, exosomes, nanoparticles or viral vectors.^509^ It is hoped that with the progress of research, more ncRNAs can be approved for clinical treatment.

### Challenges of HH-targeted therapies

At present, the selective Smo inhibitors vismodegib and sonidegib have been approved by FDA for clinical treatment for cancers. However, the acquired drug resistance of cancer patients to vismodegib proves the clinical limitation of targeting Smo. Therefore, new inhibitors of HH signaling pathway have been developed to overcome this drug resistance, such as taladegib, LEQ-506 and TAK-441. Their effectiveness and safety need to be further validated in clinical trials. Furthermore, some natural compounds can affect HH signaling transduction through Smo, Gli1, Sufu and related factors. For example, vitamin D3 has entered clinical trials to evaluate its effect on non-Hodgkin’s lymphoma. Yet, these natural compounds have not been proved to be direct targets of HH signaling pathway. This can be further determined by using detailed in vivo and in vitro models. In addition, the bioavailability of many natural products is relatively low, and the concentrations used in in vitro studies cannot be achieved under physiological conditions. Therefore, it is necessary to determine the blood level at which natural products act. NcRNAs can mediate HH signaling pathway by changing Gli1/2, Smo and Ptch1 expression. Nanobased drug delivery systems are developed to deliver oligonucleotide inhibitors, such as liposomes, exosomes, nanoparticles and viral vectors. With the progress of these studies, it will be more conducive to targeted treatment of cancers with ncRNA. Finally, HH signaling transduction pathway also cooperates with other carcinogenic pathways, such as PI3K, MAPK, KRAS/BRAF and TGF-β, etc. It is beneficial to use HH signaling pathway inhibitors as combination or adjuvant therapy in cancer. Existing clinical trials have focused on the combination of HH signaling pathway inhibitors with targeted drugs of other pathways. As such, a future goal should be to define the specific function of drugs targeting HH signaling pathway, including their in vivo effectiveness, safety, side effects, etc. A deeper understanding of the mechanisms of HH-targeted therapies will prompt new treatments and continue to offer potential therapeutic targets for cancer.

## Conclusion and future perspective

Early literature primarily focused on defining the function of HH signaling pathway in embryonic development. Recent studies have begun to reveal that the orchestration of HH signaling is critical for healthy cell function. Dysregulation of HH signaling pathway is associated with the development and progression of numerous cancers. Therefore, understanding the integrative nature of HH signaling pathway has opened up the potential for new therapeutic targets for cancer. A variety of drugs targeting HH signaling pathway have been developed, and some of which have been approved as for clinical treatment for cancer. Notably, most research of the inhibitors targeting HH signaling pathway are still in the early stages. The results of several clinical trials are unsatisfactory, owing to the fact that the efficacy and safety of the drugs are not well documented in preclinical studies. Meanwhile, certain drugs targeting HH signaling pathway show limited specificity, which is due to the lack of sufficient studies to verify the mechanism of the specific action on HH signaling pathway. As implied above, with new therapeutic targets being identified at a rapid rate, a more comprehensive understanding of the mechanisms of HH-targeted therapy will solve the existing problems in the application of these drugs and pave the way for a new clinical paradigm.

In summary, our review comprehensively summarizes the function of HH signaling pathway in tissue homeostasis and cancer development. Importantly, we discussed the current application, advantages and challenges of HH signaling pathway targeted therapies, providing valuable insight and important implications for the exciting translational innovations in the future.
